# Review of Highly Mismatched III-V Heteroepitaxy Growth on (001) Silicon

**DOI:** 10.3390/nano12050741

**Published:** 2022-02-22

**Authors:** Yong Du, Buqing Xu, Guilei Wang, Yuanhao Miao, Ben Li, Zhenzhen Kong, Yan Dong, Wenwu Wang, Henry H. Radamson

**Affiliations:** 1 Key Laboratory of Microelectronic Devices & Integrated Technology, Institute of Microelectronics, Chinese Academy of Sciences, Beijing 100029, China; xubuqing@ime.ac.cn (B.X.); wangguilei@ime.ac.cn (G.W.); kongzhenzhen@ime.ac.cn (Z.K.); dongyan2019@ime.ac.cn (Y.D.); wangwenwu@ime.ac.cn (W.W.); 2 Institute of Microelectronics, University of Chinese Academy of Sciences, Beijing 100049, China; 3 Research and Development Center of Optoelectronic Hybrid IC, Guangdong Greater Bay Area Institute of Integrated Circuit and System, Guangzhou 510535, China; liben@giics.com.cn; 4 Department of Electronics Design, Mid Sweden University, Holmgatan 10, 85170 Sundsvall, Sweden

**Keywords:** III-V on Si, heteroepitaxy, threading dislocation densities (TDDs), anti-phase boundaries (APBs), selective epitaxial growth (SEG)

## Abstract

Si-based group III-V material enables a multitude of applications and functionalities of the novel optoelectronic integration chips (OEICs) owing to their excellent optoelectronic properties and compatibility with the mature Si CMOS process technology. To achieve high performance OEICs, the crystal quality of the group III-V epitaxial layer plays an extremely vital role. However, there are several challenges for high quality group III-V material growth on Si, such as a large lattice mismatch, highly thermal expansion coefficient difference, and huge dissimilarity between group III-V material and Si, which inevitably leads to the formation of high threading dislocation densities (TDDs) and anti-phase boundaries (APBs). In view of the above-mentioned growth problems, this review details the defects formation and defects suppression methods to grow III-V materials on Si substrate (such as GaAs and InP), so as to give readers a full understanding on the group III-V hetero-epitaxial growth on Si substrates. Based on the previous literature investigation, two main concepts (global growth and selective epitaxial growth (SEG)) were proposed. Besides, we highlight the advanced technologies, such as the miscut substrate, multi-type buffer layer, strain superlattice (SLs), and epitaxial lateral overgrowth (ELO), to decrease the TDDs and APBs. To achieve high performance OEICs, the growth strategy and development trend for group III-V material on Si platform were also emphasized.

## 1. Introduction

As the big data is coming, continuing rapid development of Internet business, communication network moves toward the direction of high speed and large capacity. To meet the data information transmission requirements of efficient, speedy, and integrated data, very large-scale integrated circuits (VLSI) were developed via continuing miniaturization of the transistor characteristic size according to Moore’s law [1]. Si is always considered as the backbone material in the micro- and nano electronic industry owing to its natural abundance, high mobility, larger wafer size, low cost, and mature manufacturing technologies, etc. [2]. However, as the device characteristic size reaches to the sub-7 nm technology node, Si based integrated circuits are suffering from the physical and technological limitations in speed, power consumption, integration, and reliability, which further affect the device performance [3]. At present, two main technical roadmaps were expected to prolong the Moore’s law: (I) “non-silicon” high-mobility materials, such as SiGe, Ge, GeSn, GaAs, InAs, and InGaAs, were gradually extended into CMOS technology; (II) Si-based OEICs were proposed to integrate both photonic devices (such as the laser, optical modulator, optical waveguide, and photodetector) and electronic devices (transistors) on the sole Si wafer, which owns the advantages of faster transmission speed, larger transmission capacity, and low power consumption [4].

For high-mobility “non-silicon” materials, group III-V semiconductors can provide higher electron mobility (electron mobility of GaAs and InAs can reach up to 9000 cm^2^/(Vs) and 40,000 cm^2^/(Vs), respectively), and are ideal channel material for ultra-high speed and low-power devices, such as the high electron mobility transistor (HEMT) [5,6]. For example, to overcome the downscaling limit of conventional CMOS technology, monolithic integrations of various III-V devices, such as the sub−80 nm E–mode InGaAs/InAs HEMTs [7], InP-based HEMT [8], and AlGaN/GaN HEMT [9], have been proposed, enabling dense three-dimensional (3D) integration, low-power consumption, and high-speed applications [10]. On the other hand, for Si-based OEIC, the Si-based light source is the ultimate obstacle to achieve owing to the fact that Si is an indirect band-gap semiconductor material, and its emission efficiency is very low, which makes it unavailable as the active gain medium for Si-based high-efficient light sources. In contrast, most group III-V materials are definitely suitable for the optoelectronic devices in light-emitting/absorbing devices, including light-emitting diodes (LEDs), lasers, and detectors [11,12,13,14], owing to their direct bandgap properties, indicating their stronger photon emission and absorption efficiency in comparison than indirect semiconductors such as Si, Ge [15,16], and GeSn [17]. Thus, taking advantage of the excellent properties of III-V compounds, Si-based III-V CMOS devices and III-V photoelectric devices can further greatly improve the data transmission speed and amount, which effectively reduce integrated electricity and power consumption [18]. 

To realize the monolithic integration of III-V devices on the Si platform, it is critical to develop the heteroepitaxy technique for group III-V materials on Si [19]. Growth challenges for high-quality III-V heteroepitaxy on Si will cause APBs and TDDs/cracks [20,21]. APBs are caused by a polarity difference between III-V material and Si (surfaces for the III-V material and single Si are polar and non-polar), suggesting that it is prerequisite to prevent the formation of APBs. In case the APBs nucleated at the interface between III-V and Si, which can propagate through whole III-V epilayer, this leads to the devices’ manufacturing on Si an impossibility [22]. Another important issue is the TDDs, an issue which is attributed to the mismatch of the lattice constant and thermal expansion coefficient between group III-V material and Si. As a result, both APBs and TDDs can lead to surface roughness, which act as the nonradiative recombination centers and leakage current to destroy the device performance [23]. Hence, the defects management strategy was proposed to decrease the TDDs and APBs for group III-V material, thus improving the device performance. Wafer bonding technologies, such as adhesive bonding [24,25], direct bonding [26], and fusion bonding [27,28], were adopted to form the advanced heterogeneous integration substrate platform. However, wafer bonding induces a high manufacturing cost and low integration density [29]. In addition, it is difficult to realize the graphics technology of alignment and passive devices in subsequent processing [30]. In this regard, growing high-quality III-V semiconductors on Si is the key pathway towards monolithic integration of III-V devices on Si-based OEICs.

The purpose of this review article is providing the types of defects and the mechanism of defects formation in silicon-based III-V heteroepitaxy and the detect solution. Particularly, we update recent advances in the epitaxial growth of large lattice-mismatched III-V materials on Si substrates, especially for GaAs and InP, which are both important materials for optic-device applications. This paper is arranged as follows: Section 2 introduces the fundamental challenges in III-V hetero-epitaxy on the (001) silicon wafer, and we also highlight their defect formation mechanism. Section 3 provides a brief review of growth strategies for the defect solution, including the miscut substrate, buffer layer, Strain super-lattice layers (SLSs), Aspect ratio trapping (ART), and epitaxial lateral overgrowth (ELO). Section 4 elaborates on recent approaches on growing high-quality III-V materials on Si. This includes global hetero-epitaxial thin film growth and selective-area hetero-epitaxy. Finally, we summarize the current status and discuss the potential future of III-V-on-Si heteroepitaxy.

## 2. Basic Challenges of III-V Hetero-Epitaxy on Si (001)

**Figure 1:**
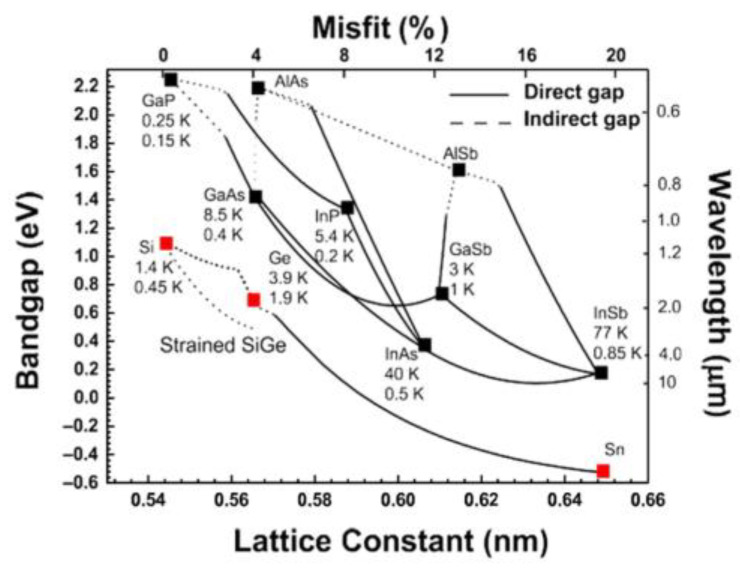
Bandgap (wavelength), lattice constants (lattice misfit), and mobilities for the most commonly used group III-V and group-IV materials. Reprinted with permission from ref. [32]. Copyright 2014 Springer Nature.

Heteroepitaxial growth represents a growth where materials with different lattice constants are grown in a stacked order, which is usually named “metamorphic growth” [31]. The relaxed lattice constant of the epitaxial layer is generally different from that of the substrate. To grow high-quality III-V layers on Si, fundamental challenges, such as the lattice mismatch, thermal mismatch, and substrate polarity difference, are the main limitations. Figure 1 shows the bandgap (wavelength) and lattice constants (lattice misfit) for the most commonly used group III-V and group-IV materials [32]. Below each semiconductor material, there are also annotation numbers for their own electron and hole mobilities, from which we can see that III-V semiconductor materials own higher electron mobility than Si, which are more suitable for the high mobility CMOS device. Meanwhile, direct bandgap property of III-V semiconductors made it more conducive to optoelectronic devices compared to the indirect gap of IV materials. However, there is a huge challenge to grow the III-V layer on the Si substrate owing to the highly mismatched nature of III-V and Si. In III-V semiconductors, GaAs (4.1%) and InP (8.0%) have close lattice constants to IV relatively, especially the Ge, which are more likely to realize the heteroepitaxy on the Si substrate. In addition, Ge has the close lattice constant and thermal expansion coefficients to GaAs, which are often used as a buffer layer to grow III-V on Si. This huge mismatch can bring out many defects such as: APBs, TDDs, stacking faults. In this section, the definition of mismatch and the mechanism of defect caused by mismatch will be introduced.

**Figure 2:**
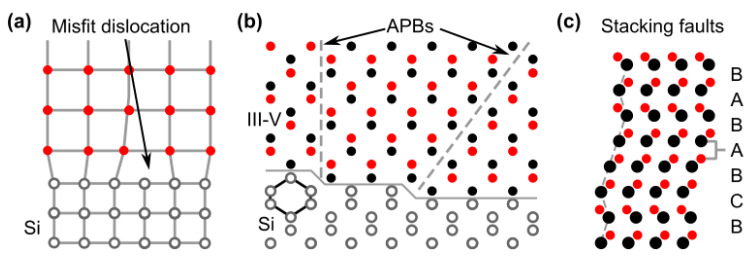
Schematic diagram of three defects (**a**) Misfit dislocation due to lattice mismatch, (**b**) APB at atomic steps of the substrate, (**c**) Stacking faults in the III-V material.

Electrical and optical properties of a semiconductor heavily depend on the crystal quality and, hence, defects in the crystal structure. There are several types of defects that can occur in semiconductor crystals, such as structural defects or compositional defects. Considering the spatial extension as a criterion, defects can be classified as 0 D point defects, such as vacancies, 1D line defects, such as misfit dislocations (MDs) or threading dislocations (TDs), 2D planar defects, such as APBs and stacking faults, 3D defects, such as voids. A detailed overview of defects is given by [33,34]. Figure 2 depicts three defect types relevant in this work. Figure 2a depicts a misfit dislocation forming at the interface to compensate for different lattice constants of the materials. Figure 2b shows the APBs’ defect. Homopolar III-III or V-V bonds can form due to the atomic steps grown on the non-polar Si substrate, which lead to the formation of APB. Figure 2c is the stacking faults that can occur during the III-V growth. If the stacking sequence changes in every layer, a zinc-blende (ZB) ABC stacking can be switched to a Wurtzite (WZ) ABAB stacking [35,36], which can impact the optical band gap since some semiconductors exhibit different band gaps for different crystal structures [37] or even change the band gap from indirect to direct or vice versa [38,39]. The heteroepitaxial growth of mismatched III/V on Si introduces additional challenges; hence, the mechanisms of challenges and the defect will be discussed below. 

### 2.1. Lattice Mismatch

One important source of strain in heteroepitaxy is the difference in the lattice constant between different materials, referred to as lattice mismatch. This mismatch introduces strain in the epitaxial layer since it is forced to adapt to the lattice constant of the substrate when it is being deposited on. Eventually, after exceeding a critical thickness, the energy stored as strain will become so huge that the layer will relax. For example, at room temperature, the lattice constants of Si and GaAs were 0.543 nm and 0.565 nm, respectively, and the lattice mismatch was 4.1%. The strain in the heteroepitaxial layer resulting from mismatch is given by [4]:

(1) $$\alpha_{m}=\frac{\alpha_{s}-\alpha_{o}}{\alpha_{o}}$$

where *α_m_* is the mismatch strain in the epilayer; *α_o_* and *α_s_* are the substrate and overlayer lattice parameters, respectively.

If *α_o_* is greater than *α_s_*, it is a tensile strain; otherwise, it is compressive strain. In an epitaxial layer grown on a foreign substrate, the layer is subjected to biaxial strain in the plane of the substrate (normally the (001), if it is unrelieved, the biaxial strain will translate to a strain in the vertical direction according to:

(2) $$\varepsilon^{⊥}=\varepsilon^{\|}\frac{1}{R_{B}}=\varepsilon^{\|}\frac{C_{11}}{2C_{12}}$$

### 2.2. Thermal Expansion Coefficient Mismatch

Most materials not only have specific lattice constants but also specific coefficients of thermal expansion (CTE). This is highly relevant in heteroepitaxy since epitaxy is normally carried out at a temperature of several hundreds of degrees, which means that the lattices of two different materials will contract to different extents upon cool-down. Going from growth temperature to room temperature, there will be an amount strain introduced in the epitaxial layer according to [40]:

(3) $$\varepsilon_{th}=\int_{T_{G}}^{RT}\alpha_{S}-\alpha_{0}dT$$

where *α*_s_ and *α*_0_ are the thermal expansion coefficients of the substrate and the epitaxial overlayer, respectively, and *T_G_* the temperature at which growth takes place. Since the grown layer is normally more or less relaxed during growth, the introduced thermal strain may lead to formation of dislocations. 

When III-V thin film is deposited on a thick substrate, the layer will undergo a formation of misfit dislocations and threading dislocations. After the growth is completed, during the process of lowering the temperature of the wafer, the difference in CTE between the two causes the shrinkage ratio of the two materials to be different, resulting in thermal strain. We assume that during the growth process, it is completely relaxed. After the wafer is cooled down to room temperature, larger CTE (III-V materials) causes greater contraction than the Si substrate, so tensile strain is generated in III-V epitaxy. Nevertheless, the strain caused by the thermal expansion mismatch can be solved through buffer thickness. However, the thermal cracks emerge easily if a thick buffer accumulates too much strain energy when temperature changes. For example, CTE of GaAs (6.6 × 10^−6^ K^−1^) is larger than Si (2.3 × 10^−6^ K^−1^); the thermal mismatches between Ge, GaAs, and Si are 103%, 105%. Thickness for III-V films on Si is typically below 10 µm [41]. Therefore, huge thermal strain is generated in the thick III-V layer when the temperature drops to room temperature, resulting in thermal cracks through the III-V epitaxial layer. Similar to other defects, the presence of thermal cracks introduces destructive effects on the quality of the III-V epilayer and performance of optoelectronic devices, such as light scattering centers, the electrical leakage path, and a limitation on the total thickness of the epilayer [19]

### 2.3. Anti-Phase Boundary

Most materials have their own crystal structure and surface primarily. The V group (Si, Ge) has a diamond crystal structure, while III-arsenides and III-phosphides have a zincblende crystal structure which makes the different types of atomic stacking. For example, the diamond crystal structure has its ABAB…atomic stacking, but the zincblende crystal structure has its ABCABC…atomic stacking. When the III-V layer is grown on the Si substrate, the different types of atomic stacking make the APB defect formation, which arises from the polar on nonpolar nature of the III-V/Si heteroepitaxy and monatomic step of the (001) Si surface [42]. For instance, in the GaAs zincblende structure without defects, Ga atoms should be alternately connected with As atoms. Once the coordination of some atoms in the structure changes so that Ga atoms are no longer connected with As atoms, a two-dimensional structural defect will be formed at the interface where the changes occur, named APB. APBs arise as the existence of steps with odd atomic thickness on the surface of element semiconductor substrates (Si or Ge) and the uneven coverage of group III or V sources during silicon surface pretreatment [43]. 

**Figure 3:**
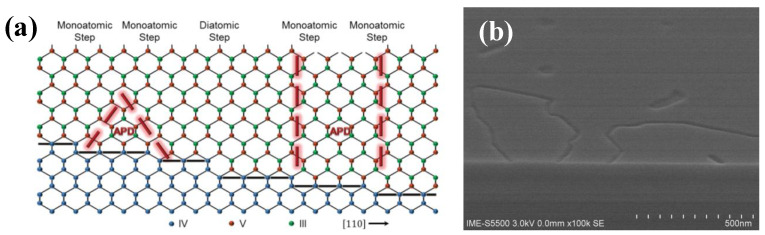
(**a**) (Color online) Schematic showing nonpolar/polar interface between Ge and GaAs. Monoatomic steps on the Ge surface result in APBs, planes of As-As, or Ga–Ga bonds. The APD can either self-annihilate (left) or rise to the surface (right). Diatomic steps on the Ge surface (center) do not result in APD formation. Reprinted with permission from ref. [44]. Copyright 2016 American Vacuum Society. (**b**) SEM plan view images of GaAs/Ge/Si (100) sample with APBs. Reprinted with permission from ref. [47]. Copyright 2021 Springer Nature.

In the process of substrate processing, it is impossible to obtain the (001) substrate with a perfect crystalline orientation. In this way, there are certain atomic steps on the actual substrate surface, which is a general monatomic layer height. The causes of APBs are shown in Ge substrate epitaxial GaAs. In the metal-organic chemical vapor phase epitaxy (MOCVD) system, arsenide (As) is pretreated with an arsenic atom (ideal) to grow GaAs on the Ge substrate (001) with the mono-atomic step surface. Due to the presence of the monatomic Ge step, the As atom and Ga atom are arranged alternately in the direction of (001), and GaAs interface with two orientations, and the As-As bond and Ga-Ga bond appear above the step, forming APBs. Figure 3a shows the single-layer steps (or odd layer height steps) to produce two domains in the III-V overlayer with opposite sub lattice allocation, whereas double-layer (or even-numbered) steps do not [44]. Although APBs do not involve partial dislocations, they can still interact with TDDs [45]. APBs are regarded as the non-radiative recombination centers for the optoelectronic devices, which will reduce the life of a few carriers in the device, and increase the scattering of most carriers, thus affecting the device performance. To characterize the influence of APBs on the optical properties, photoluminesce quenching and spectral broadening were usually adopted [46].

Besides, the APBs defect can be observed under SEM or AFM. As an example, irregular and curved boundaries were clearly observed for the SEM image of the as-grown GaAs/Si(100) sample (Figure 3b) [47]. APB is a plane defect, which can prevent the manufacture of Si-based III-V devices. Therefore, achieving APB-free III-V/Si heteroepitaxy is a fundament for following III-V devices’ fabrication.

### 2.4. Threading Dislocation Density

Heteroepitaxy of III/V materials on Si substrates results in the huge strain energy, which is released in the thickness of epitaxy via the formation of MDs along the heterointerface and TDs toward the surface. Thick epitaxy can release the mismatch strain but generates a large number of line defect dislocations. In addition, because of the mismatch TEC of III-V and Si, thick III-V epitaxy also accumulates much strain energy upon temperature cool down, inducing thermal cracks that emerge easily. These thermal cracks case the defects and surface roughness in the epitaxial layer; usually the dislocation density near the interface is as high as 10^9^–10^11^/cm^2^ [48,49].

**Figure 4:**
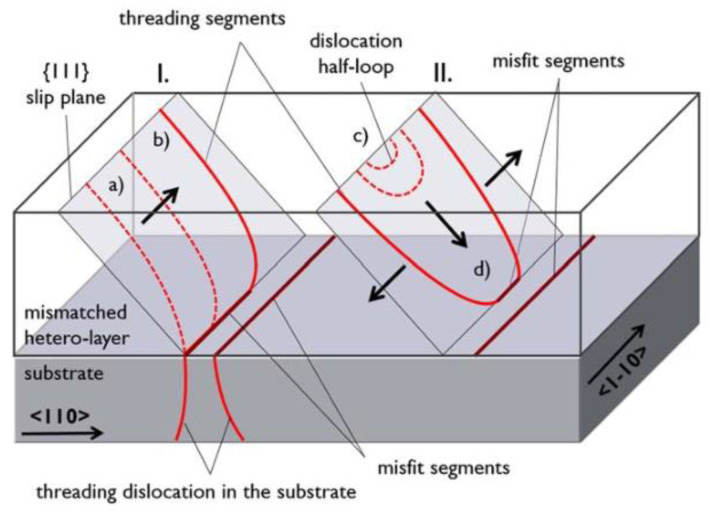
Schematic for the formation of misfit dislocation via threading dislocation glide: (**I**) TDs bend over and glide along the slip planes, (**a**,**b**) and half-loop formation; (**II**) half-loop nucleation at the surface and gliding down to the interface, (**c**,**d**). Reprinted with permission from ref. [50]. Copyright 2018 IOP Publishing.

Dislocations are line defects representing a break of symmetry along a line, called the dislocation line, which are defined by a line vector, a Burgers vector describing the distortion of the lattice along the line, and a glide plane on which the dislocation moves. Dislocations can generally be subdivided into edge dislocations and screw dislocations. The fundamental difference between these two dislocation types is that whereas the edge dislocation is perpendicular to the dislocation line vector, the screw dislocation has a Burger vector parallel to the line vector. According to the angle between Burgers and the dislocation line, 90° (edge), 0° (screw), and 60° units are the important dislocations, and the 60° unit is the main dislocation which occurs mostly at the edge of island growth during initial epitaxy. Hence, the defect formation and glide mechanism are discussed. For heteroepitaxy to begin, a two-dimensional film Tc (a few nanometers) was grown on the substrate, allowing plastic relaxation to start. Because of the lager lattice mismatch, TDs will originate from the interface and glide along the slip planes to the surface with the increase in the epitaxy. When many dislocations appear in the same area, dislocation lines are formed by upward extension of multiple obvious dislocations. The entanglement of dislocation lines changes the direction of the dislocation movement. When multiple dislocations are entangled into one, the total number of dislocation lines will decrease, thus reducing the penetrating dislocation generated by upward growth and extension. However, the dislocation entanglement generates new dislocations in different directions, some of which annihilate with epitaxial growth and some penetrate to the surface, increasing the surface dislocation density. In addition, the surface dislocation mainly consists of proliferating dislocation and penetrating dislocation, forming a “dislocation half-loop”. Figure 4 shows a sketch of MD formation by the glide of an existing TD from the substrate (I) and by dislocation half-loop formation (II). This “dislocation half-loop” has a great contribution to the strain release [50].

TDs are one-dimensional crystal dislocations in semiconductor film, which has a serious impact on the properties of semiconductors. The TD is the scattering or absorption center of the carrier or light, which reduces the free path of the electron and greatly reduces the mobility of the carrier. For example, in optoelectronic devices, TDs are the center of non-radiative recombination because the intermediate bandgap energy level in the dislocation core is highly efficient at capturing minority carriers, resulting in a minority load in the material. These defects will form a non-radiative composite center, greatly reducing device lifetime and luminous efficiency. In the case of a semiconductor laser, only a large number of minority carrier reversals are realized in the active layer to obtain an effective gain, and a laser is generated, and it is seen that the reduction in minority carrier lifetime is disadvantageous [51]. In the laser structure, if the minority carrier lifetime is reduced due to dislocations, more injected minority carriers will form a non-radiative recombination before the number of population inversions are sufficient; then, the quality of the laser will fall. Early research work pointed out that for lasers, when the TDD is exceeded, the laser will not work properly due to the reduced lifetime of minority carriers [52]. Therefore, the necessary means to prevent the dislocation from extending upward and reducing TDD in the hetero-epitaxial layer is the main problem of laser fabrication on the basis of the current stage.

TDD is a quantitative parameter which describes the quality of the epitaxial layer. It can be measured by the three common approaches: (1) Etch-pit density (EPD) measurement [53]; (2) X-ray diffraction (XRD) measurement [54]; (3) Transmission electron microscopy (TEM) [55]. In the EPD method, TDD is obtained by calculating the pits at the crystalline region by optical observation or atomic force microscopy (AFM), which is a very easy, quick, and cheap process, but it tends to underestimate the TDD. XRD provides a non-destructive measurement of TDD in the range from 10^5^ to 10^9^ cm^−2^. It is possible to calculate the TDD by measuring FWHM of rocking curve widths, because dislocations broaden the rocking curve. TEM measurement enables direct observation of TDs and quantitative analysis in the layer.

### 2.5. Stacking Faults

**Figure 5:**
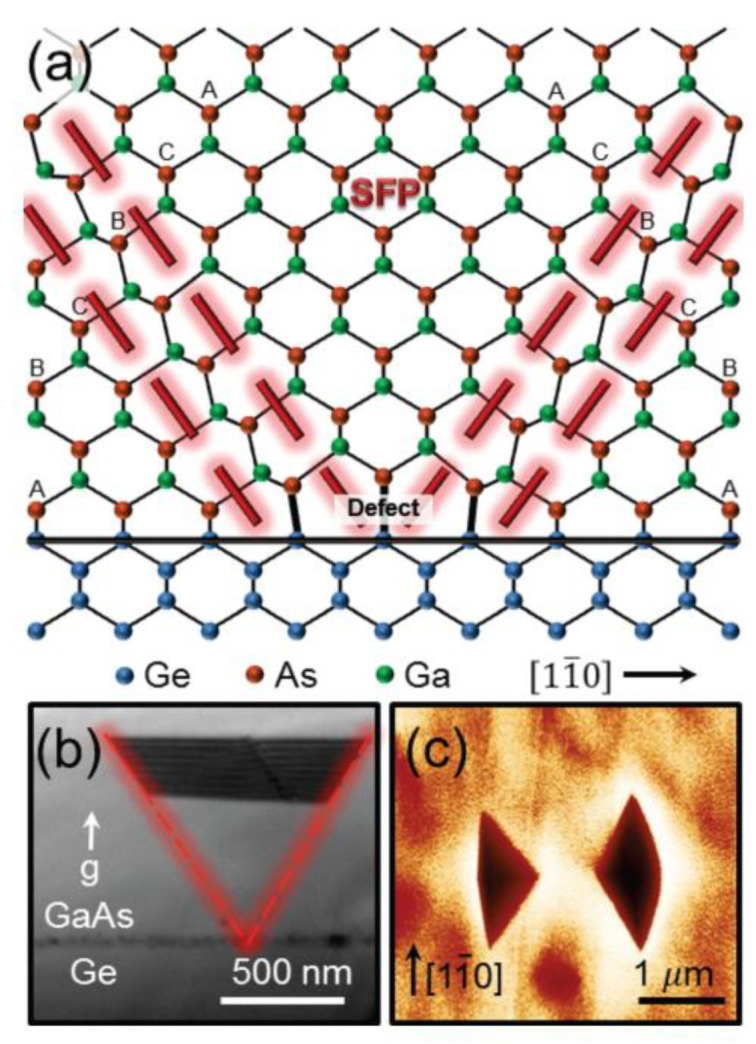
(Color online) (**a**) Schematic down [110] direction showing a SFP that originates from defect or contamination on the Ge surface; (**b)** XTEM with g = 002; (**c**) AFM image for the surface pits. Reprinted with permission from ref. [44]. Copyright 2016 American Vacuum Society.

Stacking faults (SFs) are planar defects (PDs) representing a disruption in the crystallographic stacking order. In crystals with the Face-Centered Cubic (FCC) type lattice, they normally occur on {111} planes since these have the lowest SF energy. SFs can occur either as an insertion or removal of a crystallographic plane. This may happen either during deposition or by the gliding of a plane from its natural position to another. Joseph et al. [44] investigated the SFs originating from defects or contamination on the surface prior to growth, especially at low T_sub_, which caused pits on the surface along [1$\overset{—}{1}$0] direction, as shown in Figure 5.

## 3. Defect Solution for III-V Hetero-Epitaxy on (001) Silicon Wafer

### 3.1. Surface Treatment for Si Substrate

**Figure 6:**
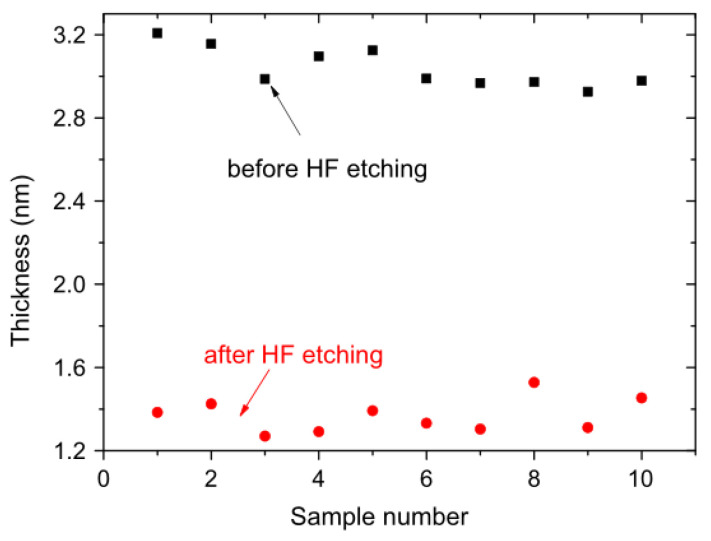
Thickness variation before and after HF 1% bath taken from a single Si substrate. Reprinted with permission from ref. [56]. Copyright 2015 Elsevier BV.

The atomic-level Si substrate platform is a basis for the III-V semiconductor devices’ manufacture. It is because rough or particle substrates can cause the stacking faults during the heteroepitaxy. To avoid stacking faults, a very clean surface for the Si substrate is very important. The ex-situ process [56] (including cycled HF dip and O_2_ plasma treatments) was developed, and film thickness variation (around 0.3 nm) is well reproduced (Figure 6).

### 3.2. Process Optimization for III-V Heteroepitaxy Growth

#### 3.2.1. Miscut Si Substrate

There are two main difficulties in heterogeneous growth of silicon-based III-V materials: APBs and TDs. As mentioned in Section 2.3, APBs’ defect arises from polar mismatch between the III-V materials and the Si substrate, two alternating (2 × 1) and (1 × 2) dimerization on the monatomic steps of the Si (001) surface. In order to avoid anti-phase disorder in the III/V layer, it is important to nucleate on a (001) Si surface with bi-atomic steps. Double steps on the Si (001) surface are desired in order to suppress APBs in subsequently grown III-V epilayers. At present, it is universally acknowledged that the use of miscut Si substrates with various angles from 2° to 6° is effective in suppression of the formation of APBs [57,58]. A miscut Si substrate can make the Si-Si dimmers parallel to the upper terrace, and the double-atomic steps can form predominantly. The step structures of Si (001) and their energetic were studied theoretically by Chadi [59]. To obtain miscut substrates, thermal treatment is usually adopted to initiate the double-step formation, which was verified on Si substrates with a miscut in <110> directions [60]. The high-temperature treatment in As atmosphere using the miscut substrate can make the surface of the silicon substrate form the diatomic step, existing as (1 × 2) surface reconstruction, and the direction of the As-As dimer or Si-Si is parallel to the direction of surface step. This form is called single-domain surface, which is a stable surface structure. The III-V family layer obtained on this structure is a single-phase structure, thus inhibiting the APBs. However, the formation of a double-atomic step does not always guarantee the APB-free III-V epitaxial layer on Si. To ensure that the Si substrate surface is almost all diatomic steps or only a few single atomic steps, the crucial keys are the diatomic step validation of the Si substrate surface and process optimization. Sakamtoto et al. [61] verified the formation of diatomic steps of the Si surface by high temperature annealing and etched by anisotropy, respectively. Carved and reflection high energy electron diffraction (RHEED) are two ways to prove the Si surface formation of diatomic steps. The mechanism of mono-atomic step transformation to diatomic step transformation on the Si surface under cyclic annealing at high temperature was analyzed by Kawabe [62].

#### 3.2.2. Bulk Hetero-Epitaxial Growth of III-V Thin Films on Si Substrate

TDD is another problem originating from the large lattice mismatch between the III-V and Si substrate. The effective suppression and reduction in TDs can be considered from two directions.

(1). The buffer layer and dislocation barrier layer with a strain field structure are the common method to reduce TD because the strain field generated by them can bend the direction of dislocation extension, thus effectively reducing the penetration depth of dislocation.

Low temperature buffer layer technology is a widely used scheme for heterogeneous epitaxy of silicon-based III-V materials, which can effectively inhibit the generation of dislocation at the interface [63,64]. The low temperature (LT) buffer layer is critical to the quality of III-V materials. III-V materials are generally island nucleated on the Si surface at a low temperature, which is the key factor affecting the nucleation density. When there is high growth temperature of the buffer layer (e.g., 650 °C), nucleation density is small, and large compressive strain causes many defects in the core. In contrast, lowering the temperature decreases the migration performance of surface nucleating atoms and the initial nucleus, thus increasing the nucleation density and reducing the size of the nucleus. The relaxation of the III-V layer at the interface releases strain and reduces the defect density at the top III-V layer [65]. Inserting buffer materials which the lattice constants between Si and III-V groups is an improved method to low TDD. The buffer layer can also be a material with a component gradient or gradual component gradient. For GaAs/Si heteroepitaxy, a wide variety of methods using Ge [66], SiGe [19], GeSnSi [67], InGaP [68] were developed. Among these materials, Ge has been most widely used because of its complete miscibility with Si, well-developed Ge-on-Si growth technology, and nearly the same lattice constant with GaAs [69].

The graded SiGe component buffer layer can effectively disperse dislocation into different component epitaxial layers to obtain a high-quality top epitaxial layer. These buffer layers can provide a high surface base for III-V epitaxial growth owing to the little mismatch between them, which can improve the quality of the III-V epilayer. In addition, the control of initial nucleation conditions of the buffer layer is also the most critical part to obtain high-quality top layer materials. For example, the lattice constant of GaP is very close to that of Si. After obtaining the high-quality GaP/Si materials, the gradient layer GaAs_x_P_1−x_ can be used to obtain the transition to GaAs [70]. However, a very thick Ge buffer layer or graded SiGe buffer for III-V growth on Si causes difficulties for interconnection between the III-V Optical device and existing CMOS devices because of huge step height. Therefore, in order to obtain both lower TDD and a thin structural layer, multiplied superlattice layers are introduced.

Strain superlattice layers (SLs) commonly consist of multiple pairs of two lattice-mismatched layers alternately under compression and tension. If the thickness of each SLs’ layer is less than a certain critical thickness (30 nm), which otherwise creates misfit dislocations, each SL accommodates elastic strains caused by lattice mismatch. The strain field of SLs can bend over and force the dislocations propagating upward to move laterally toward the edge of the sample, leading to dislocation coalescence and annihilation. Note that the SLs should have enough lattice mismatch and thickness to generate strain required for bending dislocations. SLs are used to filter dislocation, and dislocation density can be reduced an order of magnitude [71] when In_x_Ga_1−x_As/GaAs and GaAs_1−x_ P_x_/GaAs SLs structures were inserted between the silicon substrate and III-V material. However, SLs will introduce new strain in the epitaxial layer, which will cause mismatching dislocations from the III-V/Si interface to slip and interact, merge, or vanish**.** These SLs can make the propagating TDs bend over to interfaces and serpentine back and forth between the different superlattice interfaces, which increases the chance of coalescence and annihilation with other dislocations.

In addition, instead of two-dimensional SLs, the self-assembled QDs can be better used as DFLs to decrease TDD. Because the strain-driven self-organized QDs produce a large three-dimensional (3D) strain field around themselves, dislocations around QDs can be bent over and annihilated in a similar way to SLSs’ DFLs. Consequently, 3D QDs islands can promote the propagating dislocations to bend over more easily due to the stronger Peach–Koehler forces [72]. Yang et al. [73] proposed and demonstrated the employment of InAs QDs as 3D DFLs in GaAs-based material. Then, Shi et al. [74] reported a four-fold reduction in density of TDs in the InP/Si system by using self-organized InAs/InAlGaAs QDs as DFLs. A number of TDs generated from InP/GaAs and GaAs/Si interfaces propagate toward the top surface, leading to the TDD of 1.3 × 10^9^ cm^−2^. However, the growth process of quantum dots has relatively high requirements. How to control the growth conditions of quantum dots, or the best quantum dots, is needed to be solved.

(2). During the process of epitaxial growth, controlling and optimizing the growth condition of the epitaxial layer are another method to decrease TDD. Heteroepitaxy growth is a complex process science as it involves issues, e.g., nucleating, temperature, thickness, annealing, so a systematic investigation for III-V heteroepitaxy on Si is necessary. For example, too high initial temperature can induce the forming of 3D islands in in initial nucleation. A high temperature annealing process [75,76] can make defects slip and disappear and too-thick epitaxy and can increase the bow on the wafer [77]. Meanwhile, another measure includes a buffer layer with thermal cycle treatment [78], and other methods have also been developed to decrease the TDD.

Currently, the heteroepitaxy of III-V materials on Si substrates consists of primarily two methods, one is the global area epitaxy and the other is selective epitaxial growth (SEG). Global area epitaxy generally includes silicon-based III-V direct epitaxy of a group of materials and epitaxy using a buffer layer. SEG is a more effective method to reduce TDD, which can limit the defects in the patterned channel, obtaining a high quality III-V heteroepitaxial layer.

#### 3.2.3. Selective Epitaxial Growth (SEG)

Selective epitaxial growth (SEG) is introduced for the integration of different materials on the same plane or for the realization of high-quality III-V semiconductor layers. This technique is based on a certain purpose graphed Si (or Ge) substrate, locally epitaxial on the III-V layer, graphed as an insulating medium (generally SiO_2_). The graphic substrate has the advantages of releasing strain caused by heat mismatch, strong repeatability, and ease of combination with other epitaxial methods, which makes it another promising method. There are two mechanisms for dislocations reduction using graphic substrates: one is aspect ratio trapping (ART); the other is epitaxial lateral overgrowth (ELO).

**Figure 7:**
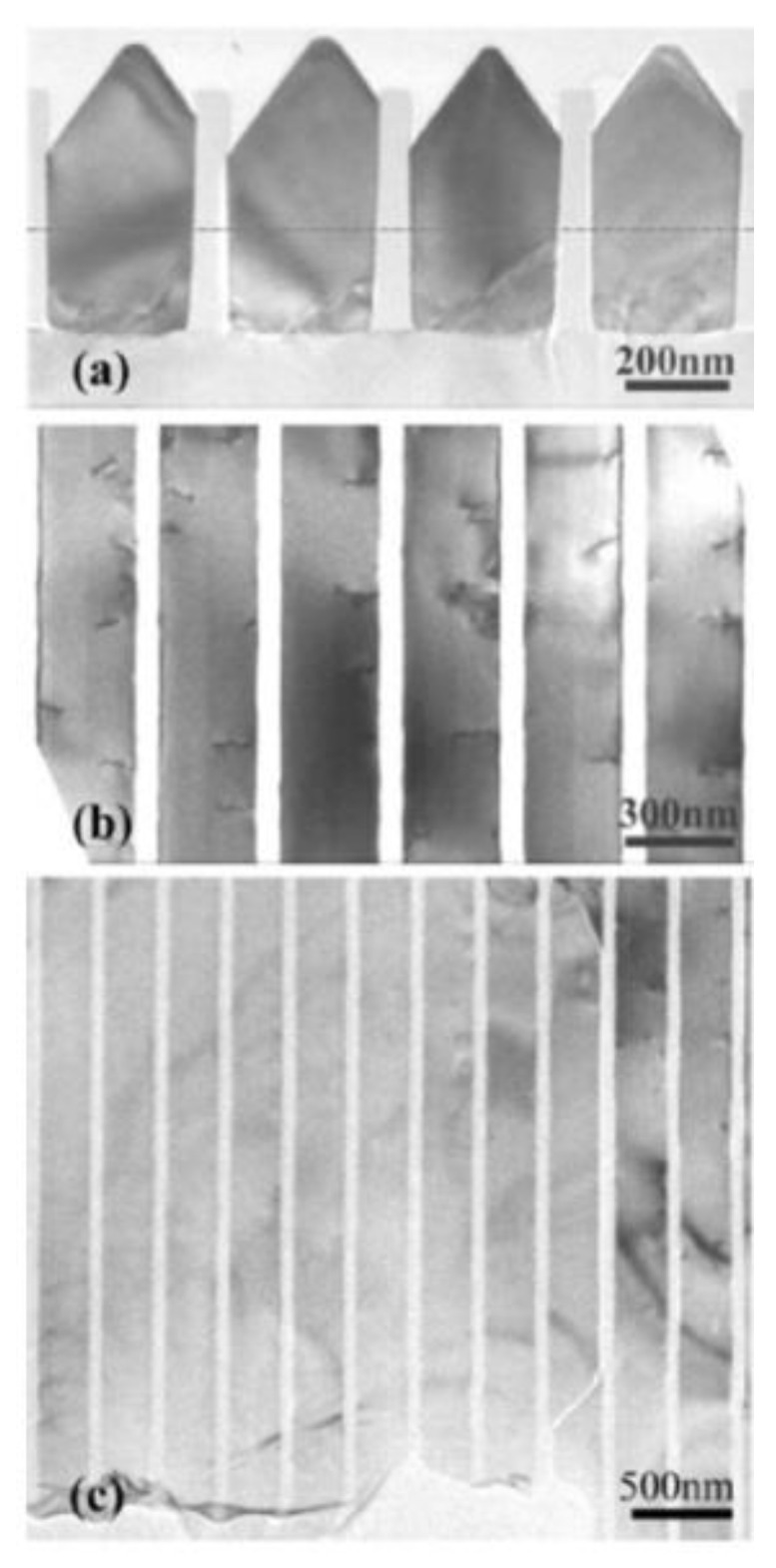
(**a**) Cross-sectional TEM of silicon-based GaAs in the groove in the direction of [110] (the groove width is 270 nm, and the depth to width ratio is 1.8); (**b**) the plane TEM image, the defect is near the insulation wall; (**c**) TEM image of the thinned sample. Reprinted with permission from ref. [79]. Copyright 2007 American Institute of Physics.

ART technology is the solution of the epitaxial high-quality III-V family layer in silicon graphics grooves with a height/width ratio greater than 2. It is a method to limit the dislocation at the bottom of the groove by using the limiting effect of the SiO_2_ side wall on the dislocation in the groove on the Si graphics substrate through the selection epitaxy. In the groove of this size, the growth plane changes from the original (001) plane to a crystal plane composed of {111} and {113} when the group III-V material was grown [79]. Defects, such as dislocations, also extend along the crystal plane and are limited when dislocations meet the groove insulation wall, thus obtaining a top layer with almost no defects (Figure 7).

ART epitaxy technology has the following advantages: (1) easy to integrate with a variety of high mobility group III-V materials and device structures; (2) it can use the selective epitaxy to achieve the epitaxy growth of Ge materials and group III-V materials between different grooves to achieve the monolithic integration of the Si base; (3) the graphic substrate of the scheme can be prepared by STI (shallow trench isolation) templates in traditional Si-based microelectronic technology, which is convenient for future large-scale integration; (4) the scheme can be directly in the groove to achieve a high-quality group III-V nano-scale on Si, compared to other nano-material preparation methods; the scheme is more convenient for the next generation of Si based high mobility device preparation and application. The scheme can combine the excellent optoelectric properties of III-V group materials with Si, and has great application potential in the future Si-based monolithic optoelectronic integration.

**Figure 8:**
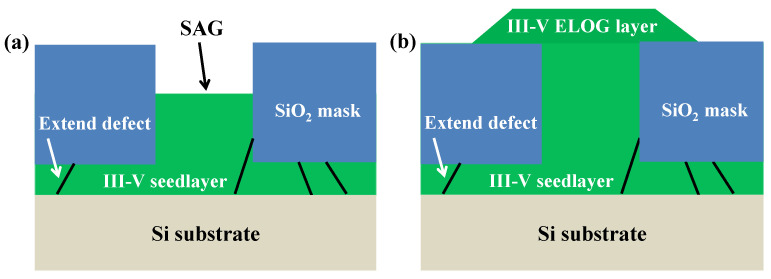
Principle of SAG (**a**) and ELO (**b**) applied to heteroepitaxy of III-V on Si.

Epitaxial lateral overgrowth (ELO) is a technique developed to overcome the difficulties with obtaining a high-quality epitaxial layer on a foreign substrate. The idea is to use a substrate of a first material with a thin layer of a second material as a starting point. The layer of second material will be full of defects due to the previously outlined mechanisms. On top of this layer, a mask, normally a dielectric such as SiO_2_ or Si_3_N_4_, is deposited, and openings in the mask are defined by lithography and etched. Growth is then conducted selectively in the openings with no nucleation on the mask (shown in Figure 8a). Once the grown material reaches the height of the mask, it starts growing laterally across the mask without nucleation on it, as shown in Figure 8b. In the laterally grown parts, propagating defects such as threading dislocations and stacking faults will be blocked by the mask and consequently cannot propagate into the layer above the mask.

It was shown that the angle between the mask openings and the crystallographic direction greatly influences the lateral and vertical growth rates as well as the bounding facet plane [80]. Recently, it was also shown that image forces acting on dislocations close to the mask sidewalls in the openings cause dislocations to bend towards the mask sidewalls, thereby enhancing the filtering effect so that virtually no dislocation propagation though the mask openings occurs [81].

Above all, traditional Si based III-V materials’ heteroepitaxy technology described above is still facing a series of problems. For example, the demand of TDD values should be lowered to 10^−6^ cm^−2^, the RMS surface roughness as low as below 0.5 nm, and compatibility with the traditional CMOS process make it difficult to realize the large-scale integration application of Si-based III-V group devices in the future. Therefore, how to solve the defect of the highly heteroepitaxy mismatch of Si-based III-V group materials is the problem that most scholars are solving at present.

## 4. Latest Approach of Heteroepitaxy of Si-Based III-V Group Materials

### 4.1. III-V Thin Films Hetero-Epitaxial Grow on Si Wafer-Scale

Fabricating the optoelectronic devices, a high-quality structure of III-V layers grown on the Si substrate is prerequisite. However, defects such as the APBs and the TDDs, propagating from the heterointerface to the surface, seriously affect the performance of the device. Bulk III-V structure layers are the basis of larger scale optoelectronic devices’ fabricating. This section will introduce several methods to achieve TDD-lowering and an APB-free III-V layer on Si substrates, mainly focusing on global epitaxial growth on the bulk Si substrate.

#### 4.1.1. APB-Free of III-V on Miscut Ge/Si Substrates

**Figure 9:**
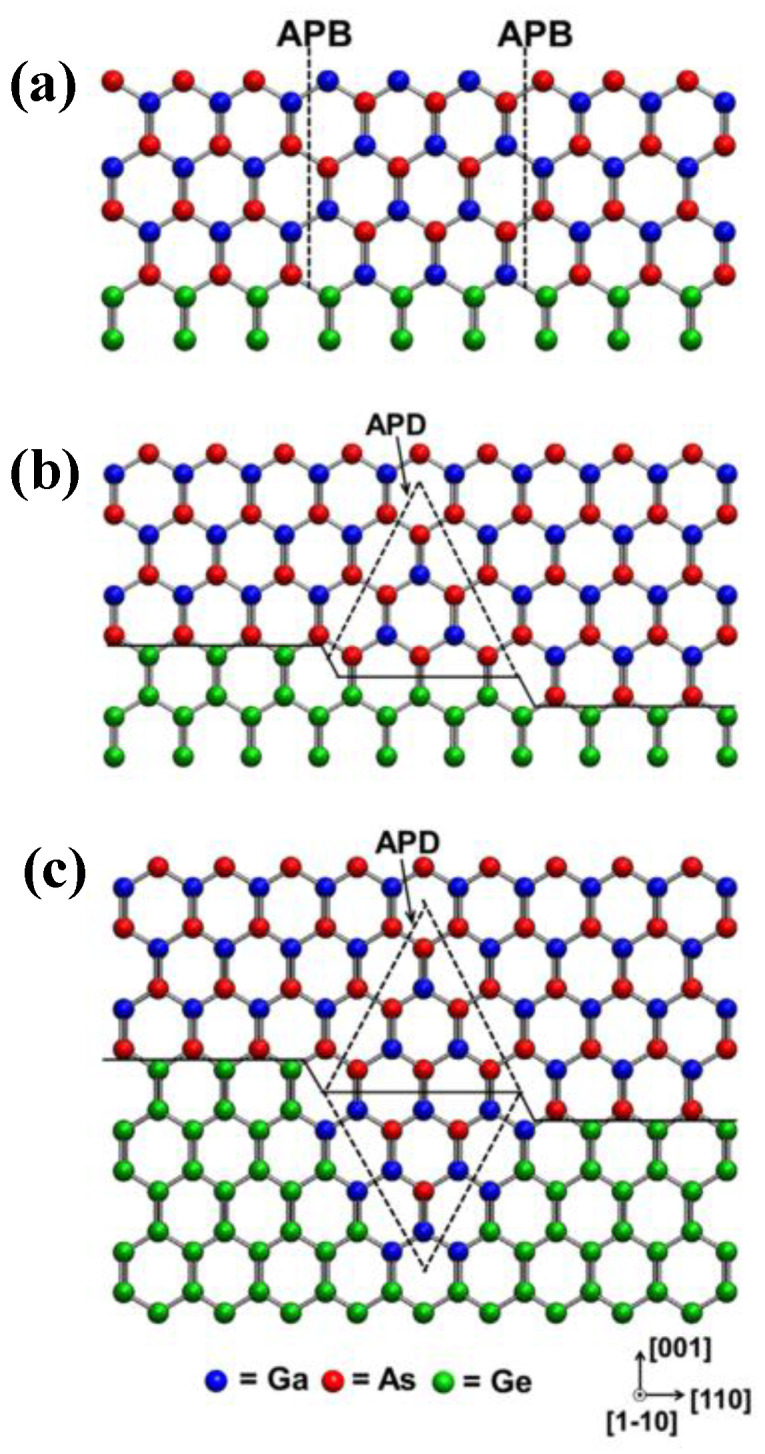
The model of generation and self-annihilation mechanisms of APBs: (**a**) an on-axis Ge (001) substrate and (**b**,**c**) miscut Ge (001) substrates. Reprinted with permission from ref. [87]. Copyright 2015 Elsevier.

The APB defect is very obvious, which is derived from the different atomic step between the III-V and Si substrate. However, substrates with a sufficient miscut exhibit a double-stepped terrace structure that can significantly reduce the APB. In 1986, Kawabe [82] grew APB-free of GaAs/Si films on a mis-oriented Si (001) substrate toward (110), which has a better structural quality and luminescence efficiency. Then, the affection of different crystal directions on APB elimination was studied [83,84]. It was suggested that mis-orientation toward (100) is optimum, since it produces steps in the vertical (110) directions, and this assists the formation of edge-type which is fit dislocations that accommodate the misfit more efficiently [85,86]. Then, the influence of different angles of the Si substrate on the inhibition of APB is also discussed. Wanarattikan et al. [87] grew GaAs layers with two-step growth on miscut Ge (001) substrates mis-oriented by big angles between 4° and 6° towards [110] direction. They found that APBs were limited at the 20–30 nm GaAs/Ge interface, while APBs-free 480 nm GaAs regions can be significantly obtained on the 6° miscut Ge (001) substrates with the RMS of 0.9 nm. A higher quality of GaAs with four times the FWHM of the GaAs epilayer than that grow on the normal Si substrate. However, the large angle substrate is not only difficult to manufacture, but also incompatible with the existing silicon manufacturing technology. Figure 9 shows the model of APBs’ generation and self-annihilation mechanisms In Figure 9a, an incomplete pre-layer at the initial Ge/Si surface induces the APBs’ generation when III-V epitaxy grows on the axis (001) Ge/Si substrate. The Ga and As atoms can be adsorbed on Ge atoms, forming Ga–Ga and As–As bonds along the [1] direction. Instead, the miscut substrate can offer a short terrace length between steps, which is conducive to APDs’ annihilation at an initial growth stage. Figure 9b,c are the model of APDs’ formation and annihilation.

**Figure 10:**
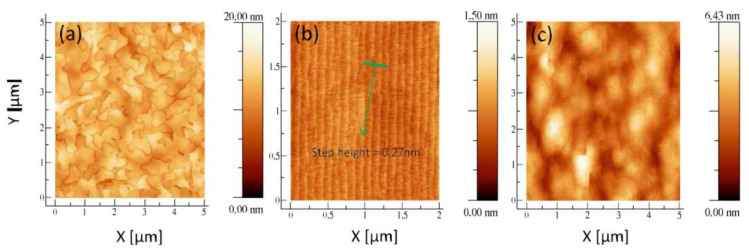
AFM image of (**a**) GaAs grown on un-optimized Si (001): High density of randomly oriented APBs; RMS roughness 1.6 nm; (**b**) 0.15° Si (001) surface after optimized preparation (800–950 °C annealing under H_2_). The surface is therefore mainly double-stepped; (**c**) APBs-free GaAs layer grown on optimized 0.15° Si (001): RMS roughness is 0.8 nm. Reprinted with permission from ref. [88].

Recently, one notably called “exact” Si (001) substrate with a slight mis-orientation (<0.5°) was made to grow APB-free III-V epilayers [88]. Figure 10 shows the GaAs layer was grown on different types of Si substrate. In Figure 10a, a high density of randomly oriented APBs on the GaAs surface with the RMS roughness of 1.6 nm was obtained when grown on a normal Si substrate. Figure 10b shows the “quasi-normal” Si (001) substrate with a 0.15° after the surface preparation procedure by annealing, presenting a 2 × 1 surface structure and predominant double steps. Based on the double steps of the “quasi-normal” Si (001) substrate, a 150 nm GaAs overlayer was deposited. Figure 10c shows the AFM image of the GaAs surface. We can see the APB-free surface of GaAs with a 0.8 nm RMS value. Above all, it is not necessary to use a large miscut substrate; instead, using this “quasi-nominal” substrate can make the GaAs layers more compatible with the existing silicon manufacturing technology.

As for the growth of InP, there is little research on bulk heteroepitaxy due to the large lattice mismatch (8%). M. Grundmann et al. [89] studied the existence or the lack of APB in the InP, providing the information about the presence of single or double atomic steps on the Si, respectively. They found the APBs-free InP could be obtained if it used the 3.8° miscut Si substrate. APBs could be decreased by the miscut substrate, but there were still hillocks on the InP layer.

#### 4.1.2. TDD-Reduction of III-V by Inserting Buffer Layers

TDD is a common problem in heteroepitaxy, which is caused by mismatch of the lattice constant and CTE between different materials. The TDDs extend directly through the epitaxy layer from the interface surface, which greatly affects the performance of devices. For a high-quality III-V layer monolithically grown on Si, achieving a low density of TD is a key issue. In particular, the TDs penetrating an active region of optoelectronic devices significantly degrade their performance.

**Figure 11:**
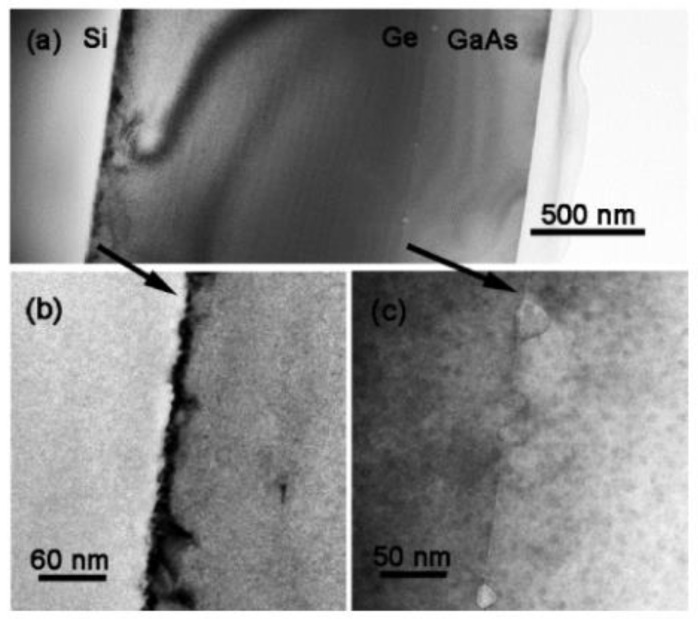
(**a**) Cross-sectional TEM images of GaAs/Ge/Si; (**b**,**c**) are the interface of Ge/Si and GaAs/Ge, respectively. Reprinted with permission from ref. [91]. Copyright 2014 IOP Publishing.

The forming of TDD begins the initial stage of III-V growth on Si, since the growth begins with the formation of the island on the Si surface. A simple two-step growth has been most widely adopted in III-V heteroepitaxy. The two-step growth starts with low-temperature (LT) growth in the initial stage, then growing the overlayer at typical high temperature (HT). During this growth method, the defects are introduced in the LT step because it can introduce a higher density of islands, which is better for islands coalescing into a continuous layer at HT growth [90]. Although the conventional two-step growth can be used in the process of heteroepitaxy, for Si based III-V heteroepitaxy, the large mismatch of the lattice constant and CET results in a large penetrating dislocation and thermal strain in the epitaxy material. Hence, a wide variety of methods have been extensively studied, including the buffer layer, annealing, three-step growth, and superlattices (SLs). Based on the III part of the growth principle of III-V heteroepitaxy on Si, the big challenge is a large lattice mismatch between them, which induces the quantitative TDD. Therefore, inserting a buffer layer in which the lattice constant and CET are matched with Si and III-V is a popular scheme of Si based III-V heteroepitaxy. This method can effectively suppress the dislocation extension from the bottom to the surface. As we know, Ge is most widely used because of its nearly the same lattice constant and thermal expansion matching between GaAs and Si. There is much research on optimization of growth process parameters. Yu et al. [64] investigated the growth of GaAs epitaxy on Si substrates with a Ge buffer. Before growing GaAs on the Ge buffer, an arsenic pre-layer was deposited with graded temperature ramped from 300 to 420 °C. Their results display that the TDD of GaAs was significantly reduced by inserting the Ge buffer. They demonstrated a graded-temperature arsenic pre-layer to improve the surface roughness to 1.1 nm, and a low V/III ratio of 20 to suppress the interdiffusion between Ge and GaAs, earning an APB-free GaAs epitaxy with the TDD of 2 × 10^7^ cm^−2^. Zhou et al. [91] also grew 450 nm GaAs films on miscut Ge-on-Si substrates by MOCVD using a two-step epitaxial method. They found that a 3 nm initial thin buffer layer is critical for the suppression of anti-phase boundaries and threading dislocations. The polishing process is essential to remove the ultrathin LT- GaAs, obtaining a smooth surface for HT-GaAs layer growth. Finally, high-quality GaAs top layers with a low TDD of 2.25 × 10^5^ cm^−2^ and the RMS less than 1 nm were obtained. Figure 11a shows the cross-sectional TEM images of GaAs/Ge/Si. Threading dislocations are restricted at the Ge/Si interface, as shown in Figure 11b. At the same time, heteroepitaxy of GaAs on the Ge surface is not the source for threading dislocation because of the little mismatch between Ge and GaAs. In Figure 11c, APBs were inhibited in the initial thin LT-GaAs buffer layer owing to the double-atomic Ge steps and high temperature annealing (>700 °C) under arsine.

**Figure 12:**
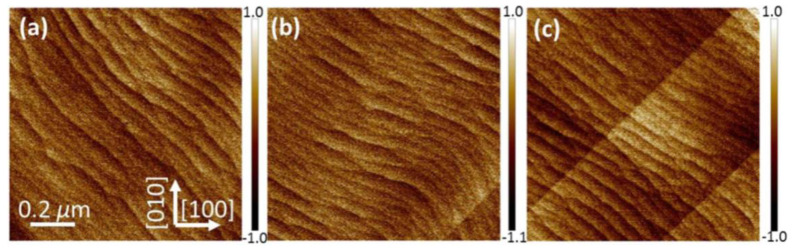
AFM images of GaAs layers grown on Ge-buffered Si (001) substrates. The Ge buffer thickness increased from (**a**) 357 nm up to (**b**) 764 nm and finally (**c**) 1377 nm. Reprinted with permission from ref. [77]. Copyright 2016 Elsevier BV.

The growth of GaAs is very sensitive to roughness and strain of the buffer layer. Therefore, it is necessary to optimize the Ge buffer layer before III-V epitaxy. Bogumilowicz et al. [77] investigated the effect of the Ge buffer layer with different thickness on the threading dislocations in GaAs epitaxial layers. First, a range of 0.36 and 1.38 μm thickness of the Ge buffer was grown on the miscut Si substrate. The results displayed that increasing the thickness of the Ge buffer results in a decline RMS value of 0.5 nm. Based on this optimized Ge buffer, a smoother 0.27 μm GaAs was obtained with a RMS less than 1 nm and low defect density of 3 × 10^7^ cm^−2^. However, a thicker Ge + GaAs epitaxial stack produced a linear increase in the wafer curvature, which causes a bow of the substrate. This bow may introduce huge strain inside the wafer, which further deteriorates the surface roughness of GaAs and the following device performance. Figure 12 shows the surface morphology of GaAs layers grown on different thicknesses of Ge-buffered Si (001) substrates. The thicknesses of the Ge buffer layer are: (a) 357 nm, (b) 764 nm, (c) 1377 nm, respectively. From the scale and the crystallographic directions, Figure 12b presents low APB density and surface roughness; the APBs’ linear density decreased rapidly as the thickness of Ge changed: 0.4 μm^−1^ for the 357 nm Ge buffer down to 0.1 μm^−1^ for the 357 nm Ge buffer and less than 0.1 μm^−1^ for 1377 nm Ge. Subsequently, Du et al. [92] also confirmed this conclusion on the influence of Ge thickness variation on the TDD of the GaAs epitaxial layer.

#### 4.1.3. TDD-Reduction of III-V by Thermal Annealing

However, the engineered Ge buffer on the Si substrate always exists with large strain, which is difficult for the following GaAs growth. Therefore, the graded Si_1−x_Ge_x_ buffer layer was used for GaAs to grow on the Si substrate, owing to offering efficient strain relaxation, and therefore a final Ge cap layer serves as a virtual substrate for GaAs growth. For a graded Si_1−x_Ge_x_ buffer grown on Si, a slow increase in Ge composition layers can induce a low number of “glissile” TDs. These effective “glissile” dislocations can increase the segment length of misfit dislocation, which accelerates the strain release. Thereby, the nucleation of new TDs is minimized. Meanwhile, the composition gradient dislocation can bend over and slip during the multilayer and then obtain the upper epitaxial layer with low TDD. Boeckl et al. [93] applied UHVCVD technology to prepare the Ge_x_Si_1-x_ buffer layer on the Si substrate, and obtained a GaAs/Si epitaxial layer with a penetrating dislocation density of 10^6^ cm^−2^ magnitude. After that, substantial efforts were devoted to achieving artificial Ge/Ge_x_Si_1−x_/Si substrates [94,95]. However, a final Ge layer of composition 100% typically takes 10 µm of epitaxial growth when it is almost fully relaxed theoretically. A thicker buffer layer will not only result in a material consumption, but also be an incompatibility with the small CMOS devices. More important, thermal strain will be introduced during the high temperature ramping down, which increases the roughness of the final product surface [96]. In addition, in order to obtain a smooth surface in rough Ge/GeSi buffers for III-V growth, a chemical-mechanical polishing (CMP) process was used, which can decrease the TDD, but increase the fabrication cost [97].

**Figure 13:**
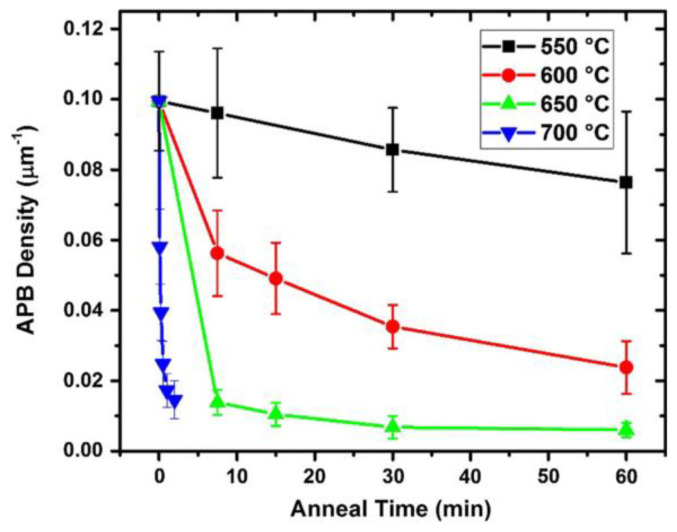
Plot of surface APB densities in the GaAs layer for different annealing conditions. APB density consistently decreases with increasing anneal time and temperature. Reprinted with permission from ref. [101]. Copyright 2019 Springer Nature.

The thermal annealing (TA) method is indispensable to reduce defect density during growth, enabling thermally activated dislocation migration and thus the annihilation of dislocations. Indeed, the TA-induced reduction of TDD in III-V/Si has been substantially investigated [98,99,100,101]. Barrett et al. [101] investigated the post growth annealing (PGA) effect on growing GaAs films on Si (001). He studied the effect of an ex situ post-growth annealing temperature range of 550–700 °C and time on the dislocation density of the GaAs layer. They found that the APB density was reduced ten times when the annealing temperature is above 650 °C. Figure 13 shows the plots of the APB density for different annealing conditions. Obviously, APB density decreases rapidly to a nonzero value after the higher temperature annealing at 650 °C and 700 °C, but for low annealing temperature, the APB density is still large even with a longer annealing time. The mechanism may be explained by the energetics of APB habit planes. High annealing temperature has sufficient energetics to propel the APB slip on {110} type planes [100].

**Figure 14:**
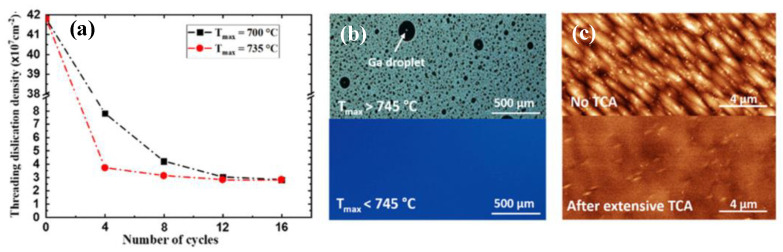
(**a**) TDD of the TCA process of GaAs-on-Si template. The minimum TDD achievable via TCA at the given GaAs thickness is about 3.7 × 10^7^ cm^−2^; (**b**) Nomarski microscope image of the GaAs surface after annealing above and below 745 °C temperature. Gallium droplets are observed when T max is higher than 745 °C; (**c**) ECCI images of the as-grown GaAs with no TCA (top) and after 16 cycles of TCA (bottom). The TDD is about 4.18 × 10^8^ cm^−2^ with no TCA and decreases to 3.7 × 10^7^ cm^−2^ after extensive TCA. Reprinted with permission from ref. [102].

Yet, previously reported annealing temperatures are either thermocouple target temperatures or ambient temperatures in the furnaces. Compared with post annealing, thermal cyclic annealing (TCA) is more conducive to defect elimination and strain relaxation. Callahan et al. [99] investigated the thermal cycle annealing (TCA) effects on the defect reduction in GaAs/Si, and reported that the dislocation density was considerably reduced to 2 × 10^6^ cm^−c^ as the annealing temperature and cycling number increased. His results revealed that the thermally induced stress as a driving force of dislocation motion contributed to the dislocation annihilation, such as coalescence. Meanwhile, based on their numerical analysis, an excellent quality of GaAs layers with a low TDD of 10^5^ cm^−2^ would be realized if the thermal cycle annealing is carried out more than 1000 times. Recently, Shang et al. [102] grew a GaAs layer through an in situ thermal cycle annealing (TCA) in the chamber to investigate the effect of TCA times on the reduction in TDD of the GaAs-on-Si template. Figure 14 shows the plot of the TDD with a different TCA process. We can see in Figure 14a that increasing the times of the TCA can reduce the TDD of the GaAs obviously, but a higher TCA of 735 °C enables a minimum TDD of 3.7 ×10^7^ cm^−2^ after 12 cycles of TCA. The mechanism is that times of TCA can prompt the TDs slip, offset or propagate to the edge of the wafer, resulting in a low thermal mismatch strain. However, a higher annealing temperature above 745 °C causes catastrophic degradation of the GaAs surface owing to the formation of a Ga droplet, as shown in Figure 14b. Figure 14c shows the comparison of ECCI images of the surface of GaAs before and after cycles of TCA. It is clearly seen that the TDD was reduced from 4.18 × 10^8^ cm ^−2^ to 3 × 10^7^ cm^−2^ after 16 cycles of TCA.

For the InP/Si, TA has also been applied to improve the crystal quality [103]. However, the effect of thermal annealing on the defect reduction is not as dramatic as in GaAs/Si because the difference in CTE between InP and Si is relatively small; thus, the dislocation motion by thermally driven strain is limited.

#### 4.1.4. TDD-Reduction of III-V by Multi-Step Epitaxial Growth

Multi-step epitaxial growth is a modified conventional two-step growth method, which inserts the Intermediate temperature (IT) layer between the LT and HT layer, and was commonly employed in recent years [104,105,106]. The two-step growth is a low temperature nucleation layer and high temperature growth layer. The purpose of the low temperature nucleation layer is to ensure sufficient time for the initial three-dimensional fusion to reduce the surface roughness and promote the fusion between the dislocations, thereby limiting the dislocation movement and reducing the penetration depth. However, the instability of the initial nucleation layer in low temperature growth makes harsh growth conditions for the high temperature growth; therefore, it is difficult to grow III-V materials stably with low surface roughness and defect density. Multi-step epitaxial growth such as three-step or four-step, which insert intermediate temperature growth, helps to prevent nuclear island forming in a metastable state from being reconstructed or damaged at high temperature. Wanarattikan et al. [87] studied the effect of the process of two-step growth and one-step growth on GaAs buffer layers using miscut Ge substrates. They designed the two-step growth with: low temperature growth at 470 °C and high temperature growth at 580 °C. Their results presented that compared with the one-step growth process at a temperature of 550 °C, two-step growth of the GaAs process exhibited a lower TDD value by about three times; the lowest APB density is 2.7 × 10^7^ cm^−2^. In following, a multi-step growth process was also studied. Wang et al. [105] demonstrate the three-step growth of GaAs on the Si (001) substrate in a low-pressure metal organic chemical vapor deposition reactor compared with two-step growth. To decrease the TDD further, TCA was also introduced for comparison. They designed their three-step growth process as: a 70 nm-thick initial LT-GaAs nucleation layer was grown at 420 °C, a 300 nm MT-GaAs epilayer grown at 630 °C, and then a 1.5-μm-thick HT-GaAs epilayer grown at 685 °C. Table 1 is different characteristic data of GaAs/Si samples grown with different procedures. Compared with the two-step growth, the TDD and RMS values of GaAs were reduced obviously by three-step growth. Meanwhile, the combination of three-step growth with two TCA steps can further improve the surface morphology and crystal quality of metamorphic GaAs. A TDD of only 1.1 × 10^7^ cm^−2^, EPD of 3 × 10^6^ cm^−2^, and the smallest RMS of 1.8 nm can be obtained via this Combinatorial method.

**Figure 15:**
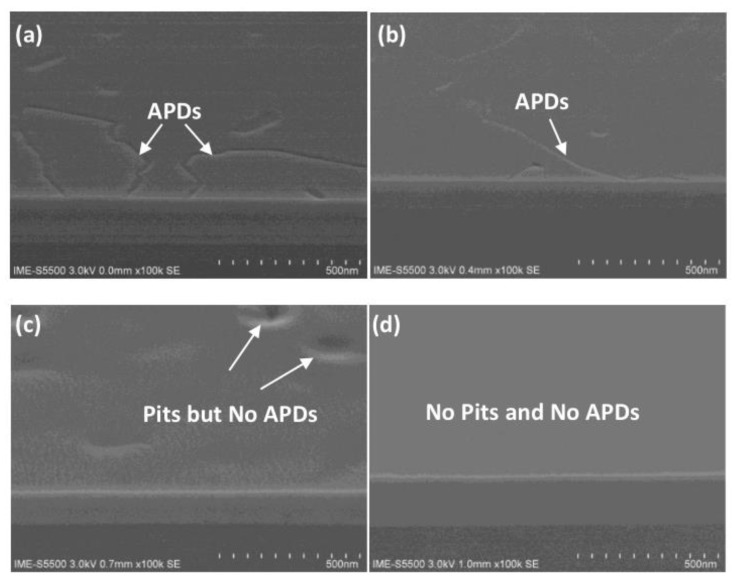
The SEM plan view images of GaAs (**a**) two-step growth on 0° miscut Si substrates and (**b**) three-step growth on 0° miscut Si substrates; (**c**) two-step growth on 6° miscut Si substrate, (**d**) three-step growth on 6° miscut Si substrate. Reprinted with permission from ref. [47]. Copyright 2021 Springer Nature.

According to the above, although three-step growth can reduce the RMS of the GaAs surface to 1.8 nm, it still cannot meet the requirements of device preparation. In 2021, Du et al. [47] also investigated the three-step growth of GaAs on both 0°— and 6°—miscut Si substrates with an engineered Ge buffer. First, a flatter Ge buffer layer was obtained through CMP, which is more favorable for GaAs growth. The conventional two-step growth process was low temperature at 460 °C, high temperature at 670 °C. The results of the two-step growth displayed a foggy surface of GaAs with the RMS of 3.4 nm. Then, an intermediate temperature at 600 °C was inserted between low and high temperature growth of GaAs to impede the defects to propagate to the HT layer. Figure 15a–d show the GaAs surface morphology of a comparison of the two-step with three-step growth on 0° and 6° miscut Si substrates. The three-step growth process can obviously eliminate the pits (TDs) on both substrates, but APB strips still exist on 0° miscut Si substrates. In other words, APB-free GaAs film with a low TDD of 7.4 × 10^7^ cm^−2^ and RMS of 1.27 nm could be obtained on 6°− miscut Si substrates by three-step growth.

For the InP/Si, direct growth of InP on Si produces a much higher TDD than that of GaAs on Si; a two-step growth is difficult to obtain a flat InP surface [107]. However, our group is developing the high-quality InP epitaxial layer on a 200 mm miscut Si platform using the multi-step growth technique, which has an APB-free InP-on-Si substrate. These breakthrough results will be submitted later.

#### 4.1.5. TDD-Reduction of III-V by Inserting Strained-Layer Superlattices Layer

**Figure 16:**
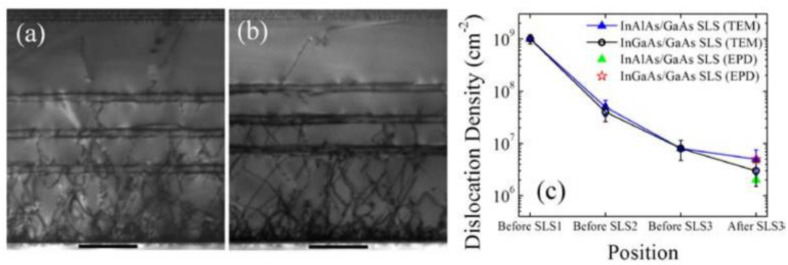
Cross-sectional TEM dark field images of (**a**) InGaAs/GaAs SLS and (**b**) InAlAs/GaAs SLS. (**c**) Reduction in dislocation induced by the SLS layers measured at different positions. Reprinted with permission from ref. [115].

Recently, strain-layer superlattices (SLs) were employed as dislocation filter layers (DFLs) to filter the dislocations’ density by bending the dislocation direction with the strong strain field around the quantum well (QW) or 3D quantum diots (QDs) [108,109,110]. The detail mechanism of dislocation being filtered and eliminated by SLs is explained in part III. Ternary-binary SLSs DFLs are widely used in III-V/Si heteroepitaxy, including InGaAsAs/GaAs, InAlAs/GaAs, InGaAs/InP, and so on [111,112,113,114]. For instance, Tang et al. [115] compared InAlAs/GaAs and InGaAs/GaAs (SLSs) as dislocation filter layers to grow 1.3 μm InAs/GaAs quantum dot laser structures on Si substrates. Two types of SLSs are designed as: five-period of 10 nm In_0.15_Ga_0.85_As/10 nm GaAs and five-period of 10 nm In_0.15_Al_0.85_As/10 nm GaAs, respectively. Figure 16a–c shows the cross-sectional TEM of low magnification of two different SLs layers. We can see that free dislocations of GaAs layers are visible after the insertion of InAlAs/GaAs SLSs in Figure 16b, but a few dislocations are also exist in GaAs layers after the insertion of InGaAs/GaAs SLSs in Figure 16a. TDD reduction of the GaAs after insertion of different types of SLSs was also characterized by TEM and EPD in Figure 16c, respectively. After three sets of InAlAs/GaAs SLSs, the GaAs sample shows an average defect density of about 2.0 × 10^6^ cm ^−2^ while the one with InGaAs/GaAs SLSs has about 5.0 × 10^6^ cm^−2^. In addition, photoluminescence (PL) also verified that the sample with InAlAs/GaAs SLSs is about two times stronger than that with InGaAs/GaAs SLSs, which means that InAlAs/GaAs SLSs are more effective in blocking the propagation of threading dislocations than InGaAs/GaAs SLSs under the similar growth conditions.

**Figure 17:**
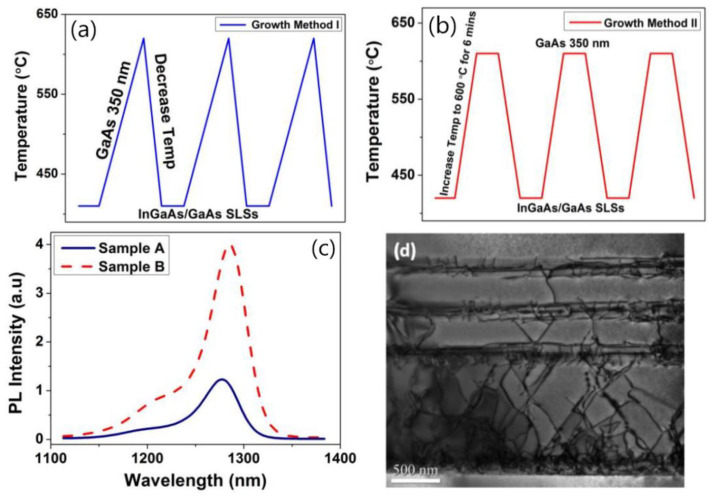
(**a**) Growth method I; (**b**) Growth method II; (**c**) PL spectra measured at room temperature for the two sample; (**d**) Dark-field cross-sectional TEM image of optimized In0.18Ga0.82As/GaAs SLSs DFLs. Reprinted with permission from ref. [116]. Copyright 2016 IEEE.

The changing of composition of SLSs materials can affect the band potential barrier, which has an important impact on defects’ limitation. Later, Tang et al. [116] investigated the indium composition and thickness of In_x_Ga_1−x_ As/GaAs SLSs for 1.3 µm QD lasers on Si. They designed the efficacy of indium composition x which were 0.16, 0.18, and 0.20, and the thickness of GaAs were 8, 9, and 10 nm. To improve the effectiveness of InGaAs/GaAs DFLs, two different growth methods were introduced in Figure 17a,b. In growth method I in Figure 17a, a GaAs spacer layer was grown during the period of heating up to 610 °C right after the deposition of InGaAs/GaAs SLSs at 420 °C. In contrast, in growth method II, the GaAs spacer layer was grown after in-situ annealing of the SLSs at 610 °C in Figure 17b. In Figure 17c, the PL peak intensity of the QD laser structure with growth method II was at least three times higher than that with growth method I. This improvement can be attributed to the high-temperature growth of the GaAs spacer layer and in-situ annealing of SLSs. It is also revealed that the optimized indium composition and GaAs thickness in SLSs were 0.18 and 10 nm, respectively. In Figure 17d, it was shown that the employment of three sets of In_0.18_Ga_0.82_As/GaAs SLSs DFLs effectively blocked and annihilated the TDs. In addition, the UCSB team [113] grew the GaAs layer on the GaP-engineered Si substrate using In_0.1_Ga_0.9_GaAs/GaAs strain super-lattices (SLSs). The In_0.1_Ga_0.9_GaAs/GaAs SLSs can further reduce the penetration dislocation density in the GaAs buffer layer to 7.3 × l0^6^ cm^2^.

**Figure 18:**
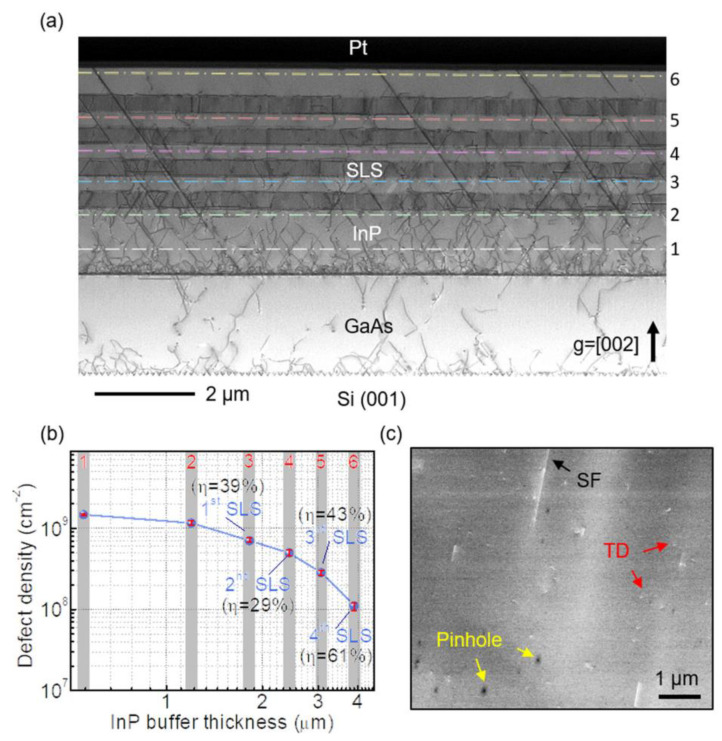
(**a**) Cross-sectional STEM image of the InP-on-Si template to demonstrate the generation and propagation of threading dislocations and stacking faults; (**b**) Extracted dislocation density as a function of the InP buffer thickness at various growth stages. (**c**) Typical ECCI image of the InP surface, where different kinds of defects can be identified and counted. Reprinted with permission from ref. [107]. Copyright 2020American Institute of Physics.

In the InP/Si platform, the DFLs based on InGaAs/InP, In(Ga)AsP/InP, (In)GaP/InP [117,118] were also commonly adopted. In 2020, Klamkin et al. [107] reported their advanced InP-on-Si virtual substrate which is optimized by inserting In_0.73_Ga_0.27_As (13 nm)/InP (19 nm) 10-pair SLSs on the GaAs-on -V-grooved Si (GoVS) template. In this report, InP buffer layers were first grown on the GoVS template using multi-step growth, followed by four sets of InGaAs (13 nm)/InP (19 nm) 10-pair SLSs with 300-nm-thick InP spacer layers. Figure 18a shows the cross-sectional STEM image of the InP-on-Si template and the extracted dislocation density at various growth stages. Six lines are the different growth stages. First, at low temperature growth of InP on the GoVS substrate, many dislocations are visible at the interface of GaAs and InP; the TDD is in the order of 10^10^ cm^−2^_._ After the three-step growth of InP (line 2), a large number of TDs are annihilated and coalesced, leading to a reduced defect density of approximately 1.5 × 10^9^ cm^−2^. In following, a higher set of SLSs is inserted to filter dislocations, which can be seen in the image that the TDs decrease after the multi-SLSs insertion. Figure 18b shows the plot of the TDD value with the various growth stages. The dislocation filtering efficiency is enhanced for the higher set of SLSs; the final InP surface TDD is reduced to 1.17 × 10^8^ cm^−2^ after four sets of InGaAs/InP DFLs (line 6). The final InP surface morphology was also characterized by ECCI in Figure 18c. APB-free and low TD were present, but few SFs and pinholes appear. The counted densities for TDs, SFs, and pinholes were 6.9 × 10^7^ cm^−2^, 1.1 × 10^7^ cm^−2^, and 3.5 × 10^7^ cm^−2^, respectively. Such pinholes are mainly due to the fact that higher SLSs also introduce new dislocations. It was revealed that InGaAs/InP SLSs can obviously reduce the TDD to 10^7^ cm^−2^, but they formed a rough surface with many hillocks.

**Figure 19:**
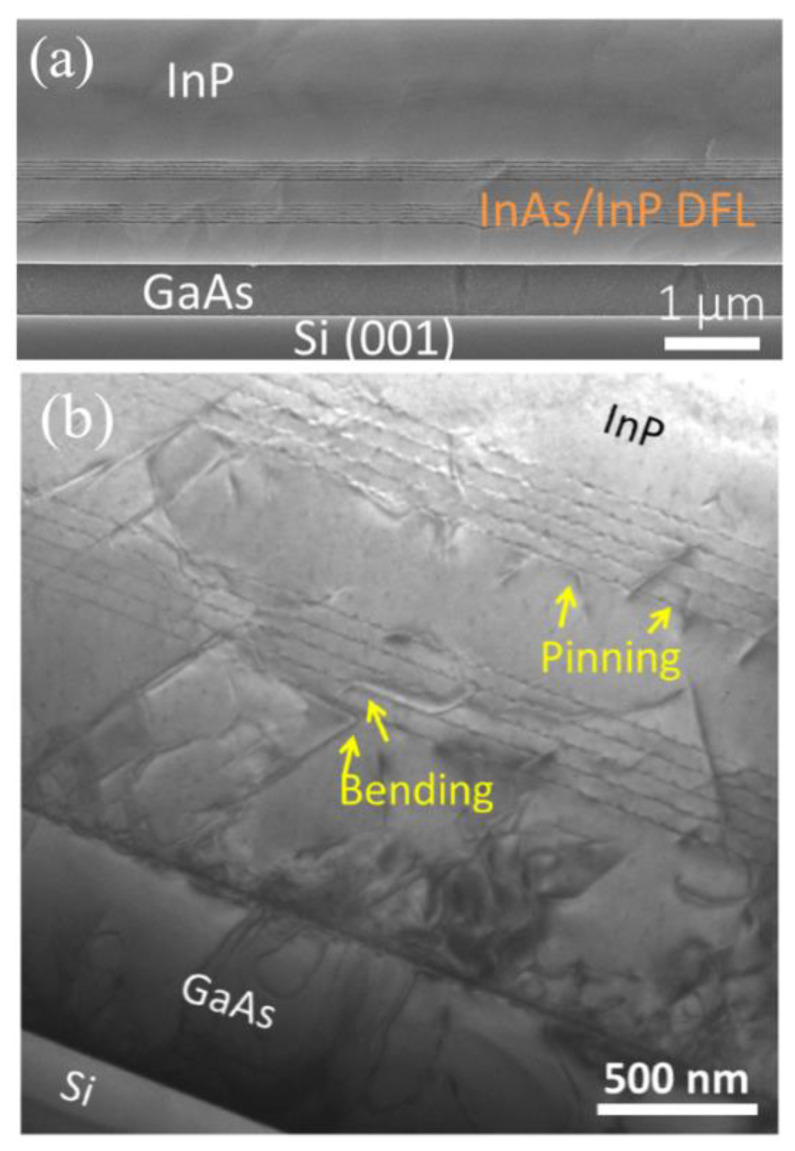
(**a**) Cross-sectional SEM of InP layer grown on planar GaAs/Si by inserting two periods of five-layer InAs/InP QD DFLs; (**b**) cross-sectional TEM images of InP grown on planar GaAs/Si by inserting two periods of five-layer InAs/InP QD DFLs. Reprinteed with permission from ref. [119]. Copyright 2018 AIP Publishing.

In addition, the self-assembled QDs can be used as DFLs to filter the TD of the InP/Si layer. Because the strain-driven self-organized QDs produce a large three-dimensional strain field around themselves, dislocations around QDs can be bent over and annihilated in a similar way to SLSs DFLs. Shi et al. [119] grew the InP layer on the GaAs-on-Si substrate by inserting optimized multiple InAs/InP QDs as DFLs. They inserted two periods of five-layer InAs/InP QDs dislocation filters to obtain a smoother surface before the subsequent QD stack growth during the HT-InP layer growth. A RMS roughness of 2.88 nm of a binary InP layer can be obtained, minimizing the generation of large InAs islands. Figure 19a shows the cross sectional of InP on planar Si inserted with two periods of five-layer InAs/InP QD DFLs. The structure of InP and the InAs/InP DFLs layer are observed clearly. Figure 19b shows the effect of InAs/InP DFLs on defect elimination by TEM images. It can be seen that the TDD is bent and eliminated by the first five-layer InAs/InP QD DFLs, but sufficient defects can still propagate upward to the top surface. After the second stage of QD DFLs, very few TDs can be detected, and most of the defects are propelled or pinned by the stacked QDs, leading to either annihilation or coalescence of the TDs. Finally, a low defect density of 3 × 10^8^ cm^−2^ was achieved for the InP-on-Si substrate.

Adopting SLSs’ dislocation filter to reduce the TDD also was used with the selective epitaxial technology recently. For instance, Norman et al. [120] obtained the GaAs/Si epitaxial layer by SEG; the In_0.15_Ga_0.85_As SLSs dislocation filter was grown on the V-groove graphic substrate. ECCI shows a low dislocation density as 2 × 10^7^ cm^−2^.

### 4.2. III-V Thin Films Selective Epitaxial Growth on Si Wafer-Scale

However, the miscut Si substrates are not popular in current industrial process flows because of the high consumption and are incompatible with advanced Si manufacturing technologies. Thus, some researchers explore other methods to reduce the problem of APBs in epitaxial GaAs layers on nominally on-axis Si (001) wafers.

Selective epitaxial growth (SEG), allowing the epitaxial layer to grow on the pre-defined region by substrate patterning, offers additional control over the strain relaxation process to control the dislocation. Aspect ratio trapping (ART) is the most common method of SEG owing to the simplicity of design. This epitaxial technology, through a high depth-width ratio, limits the dislocation and other defects originating from the Si surface to the bottom of the groove by using the SiO_2_ sidewall, so as to obtain high-quality, dislocation-free III-V materials at the top, which greatly reduces the dislocation density in the materials. The ART template can be made by STI technology from traditional CMOS processes, which can realize the monolithic integration of III-V group materials and Ge materials on the Si substrate.

#### 4.2.1. Aspect Ratio Trapping Technology (ART)

**Figure 20:**
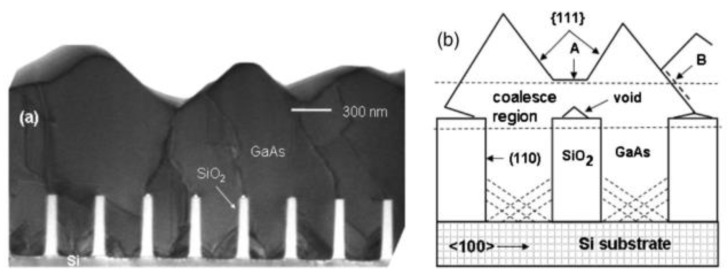
(**a**) Cross-sectional TEM image of GaAs grown on ART patterned Si; (**b**) coalesced GaAs grown and the schematic illustration of coalesced GaAs growth. Reprinted with permission from ref. [123]. Copyright 2008 American Institute of Physics.

The original concept of ART for epitaxial III-V on the silicon substrate was proposed by Fitzgerald in 1991 [121]. At that time, this idea was called the “epitaxial necking effect”: They point out that the {111} crystal plane family is the slip plane in the zinc-blende lattice structure, and the TD is mainly along the {111} plane, which develops a 45° with plane (001). So, when the width of the selected area is less than the thickness of the epitaxial material, the TD will reach the edge of the material and terminate. The method was firstly revealed in Ge/Si hetero-epitaxy [122] and then applied to III-V/Si epitaxy. Bai et al. [123] introduced the ART method directly to GaAs epitaxy on silicon. They first deposited SiO_2_ on silicon with a certain thickness and then etched along the [110] direction to reach a certain surface of the silicon substrate width of grooves. The SiO_2_ side wall of the groove limits the development of TD from the GaAs-silicon interface; part of GaAs has almost no defects, as shown in Figure 20. In Figure 20a, a lot of dislocations are visible at the interface of GaAs/Si, and gradually limited within the SiO_2_ trenches, then completely terminated within the first 200 nm of GaAs growth. The schematic of initial coalesced GaAs growth and coalesced GaAs growth is shown in Figure 20b.

**Figure 21:**
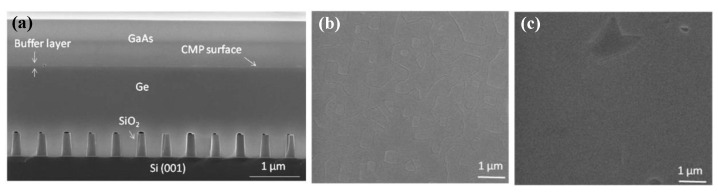
(**a**) Cross-sectional SEM image of GaAs overgrown on polished SEG Ge buffer layer on patterned Si (001) substrate. (**b**) Plane view of GaAs surface grown on exact oriented (001) Ge substrate and (**c**) on polished SEG Ge/Si substrate. Reprinted with permission from ref. [124]. Copyright 2009 Elsevier BV.

Although the above SiO_2_ mask can limit the development of TDD, the APBs are still generated, which bring defects to the materials and limits the photoelectric properties. Therefore, TDD and APB are further reduced by introducing a buffer layer. Li et al. [124] investigated the growth of GaAs layers on polished Ge/Si by selective ART. They first grew the Ge layer on the patterned SiO_2_ substrate, then deposited GaAs on the SEG Ge buffer layer. Figure 21 shows the layer structure. Their results indicated that APB-free GaAs can be obtained only on a polished SEG Ge buffer layer on the exact (001) Si substrate. Figure 21b shows the APB surface of GaAs when grown on a non-polished SGE Ge buffer layer. In Figure 21c, an APB-free of 1 μm GaAs layer was obtained with the full-width at half-maximum (FWHM) is only 140 arcsec. The significant APB reduction in the GaAs layer was attributed to the nature of SEG-based Ge growth, which results in a virtual miscut Ge surface after CMP. However, hard-to-control asymmetry of GaAs facets and the thicker structural layer are not conducive to device integration which remains a problem.

**Figure 22:**
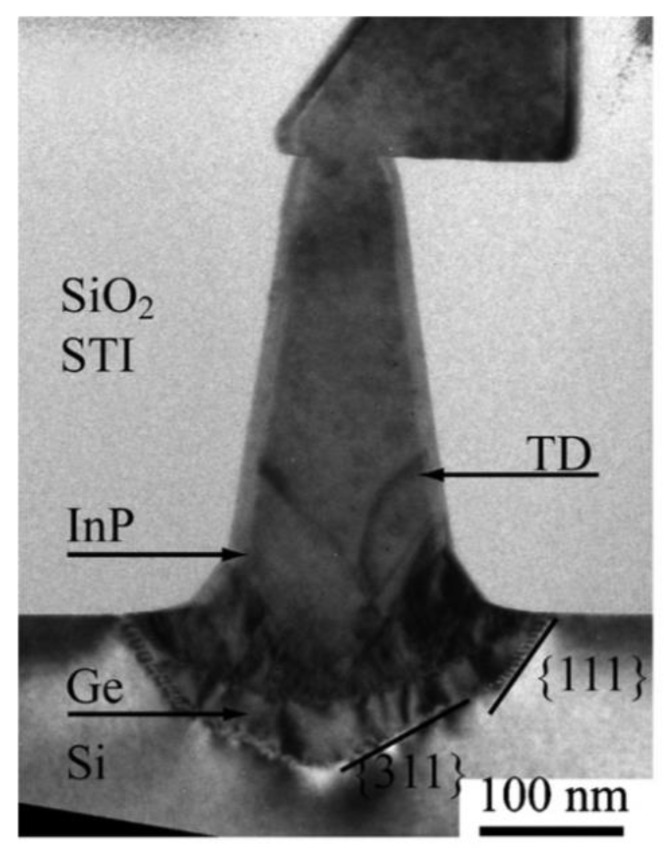
Bright field cross-section TEM images of InP grown in the 100 nm wide STI trenches. {111} and {311} Si facets were obtained after Si etch with HCl vapor. TDs are confined in the bottom of the trenches. Reprinted with permission from ref. [125]. Copyright 2010 American Institute of Physics.

In order to resolve this problem, a thin buffer layer is grown in the groove to selectively continued III-V materials. Wang et al. [125] demonstrated the SEG method of high-quality InP layers in submicron trenches on normal Si substrates using a thin Ge buffer layer. Figure 22 shows the cross-sectional TEM images of the SEG InP layer in 100 nm STI trenches. {111} and {311} facets are visible after the Si process. Then, a thin Ge buffer layer was deposited to form a relatively round surface. This rounded Ge surface removes facets, and the SEG InP grows following the Ge surface in a step flow growth mode; thus, a different crystal orientation can be avoided, which can solve the problem of voids’ formation. Meanwhile, an annealing process can prompt the single surface steps of Ge to migrate and merge into double steps, which is essential to avoid any APB formation. In addition, many threading dislocations’ TDs are confined at the side of the trench; an APB-free and low TDD InP layer is obtained at the top of the trenches.

**Figure 23:**
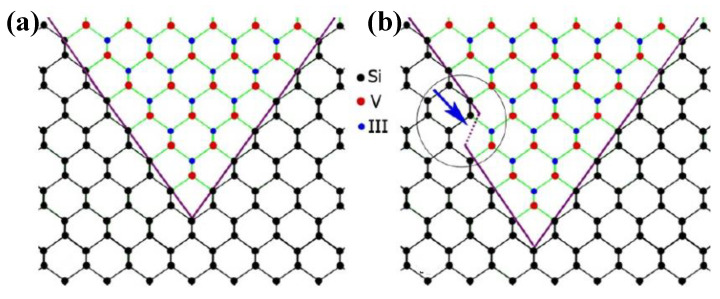
Schematic diagrams of III−V lattice in: (**a**) the “V-shape” of Si with {111} facets; (**b**) a monatomic step on a (111) plane. Reprinted with permission from ref. [126]. Copyright 2012 American Chemical Society.

Even though a pre-epitaxial Ge buffer layer is helpful to solve the APB, the quality degradation of III-V materials still cannot be completely eliminated because the diatomic steps cannot form naturally, spontaneously on the Si declination substrate and Ge buffer layer surface. Although the formation of diatomic steps can be promoted by certain pretreatment, it is still not guaranteed that all epitaxial interfaces are diatomic steps. When the III-V materials are deposited in the position without diatomic steps, there will always be possible APBs. The density of defects such as twin planes traveling along the trench direction is fairly high. In 2012, the IMEC group innovatively developed a method to construct natural diatomic step surfaces by pre-etching the silicon substrate at the bottom of the ART method SiO_2_ groove into a “V” groove consisting of two {111} faces using an alkaline solution, which can effectively suppress the generation of APB in the III-V epitaxial layer [126]. Growing III-V materials on V-grooved (111) Si surfaces can greatly enhance the quality of epitaxial III-V materials in the ART process [127,128,129,130,131,132,133,134]. There are many advantages by the use of {111} Si V-grooves in the ART growth process. First, APBs can vanish in the V-grooves by the crystallographic alignment between the Si and III-V materials; secondly, compared with the Si (001) plane, little defects will generate when III-V materials nucleate on the Si (111) plane; thirdly, it can selectively grow the active region in any location on the silicon substrate, and the size and position of the active area can be controlled manually. Figure 23 shows the schematic diagrams of the III-V lattice in the “V-shape” of Si. Figure 23a shows III-V lattice in the V-shape of Si with {111} facets along the [110] direction [125], which have the same polarity, but in Figure 23b, a single step on the Si (111) surface is equal to the interplanar spacing of Si {111} planes; such steps might not lead to the formation of APBs in the III−V material.

**Figure 24:**
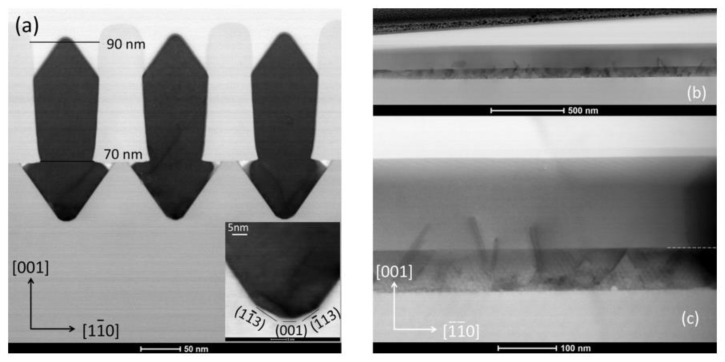
(**a**) Bright field STEM image of GaAs on V-grooved Si. Inset: High magnification bright field STEM image of the trench bottom [113] direction; (**b**,**c**) show low and high magnification bright field-STEM images of a cross section parallel to the trenches Reprinted with permission from ref. [128]. Copyright 2015 American Institute of Physics.

Tommaso et al. [128] grew GaAs fins in sub-100 nm trenches patterned on Si (001) substrates using the ART approach. They demonstrated the trench bottom geometries in “V” shaped with a consequence of the NH_4_OH etch. A 75 nm deep of the “V” shaped groove is formed with the presence of small {113} and (001) facets, which can minimize the interfacial energy and prevent the formation of APBs. Figure 24a–c display bright field STEM images of GaAs-on-V-grooved-Si in directions both perpendicular and parallel to the trenches. All TDs (meandering lines) are found annihilated on the oxide walls and confined at the trench bottom. Few {111} planar defects can be identified, and none of them reach the surface, suggesting the upper part of the inspected GaAs portion is free of defects.

**Figure 25:**
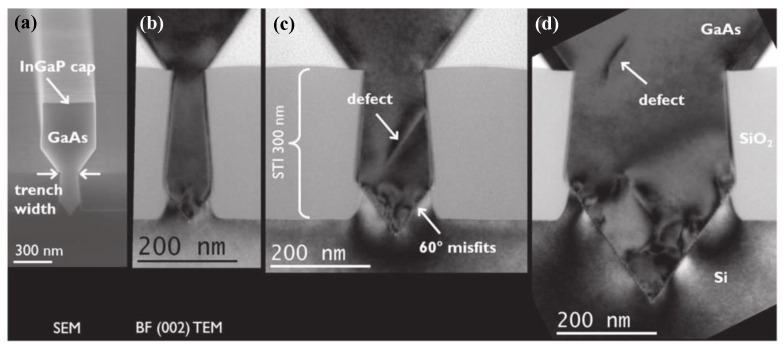
(**a**) a slightly tilted cross SEM image of a GaAs nano-ridge was deposited in 100 nm wide trenches capped with an InGaP cap; cross-sectional bright field TEM images of GaAs were grown in (**b**) 40 nm; (**c**) 100 nm; (**d**) 300 nm trench. Reprinted with permission from ref. [50]. Copyright 2018 IOP Publishing.

However, the defects of III-V material are also related to the structure of the groove and the growth process. The different aspect ratio also affects the limitation of material defects. Kunert et al. [50] reported that the GaAs fins selectively grow in a V-shaped trench with the aspect ratio of 7.5, 3, 1. Figure 25 shows a cross sectional SEM of the GaAs selectively grown in different ARs. In the case of the ARs being 7.5 and 3, all dislocation defects are trapped and confined inside the STI region in Figure 25b,c. However, for the ARs of 1 in Figure 25d, TD defects are also found above the trench, which indicates that an AR of 1 is not sufficient to block all dislocation. In fact, in these narrow trenches, the InP layer is very defective, with an extremely rough and discontinuous surface. As the surface treatment for wide and narrow trenches is identical, we must conclude that the geometrical confinement within the narrow trenches induces a transition from 2D to 3D growth.

**Figure 26:**
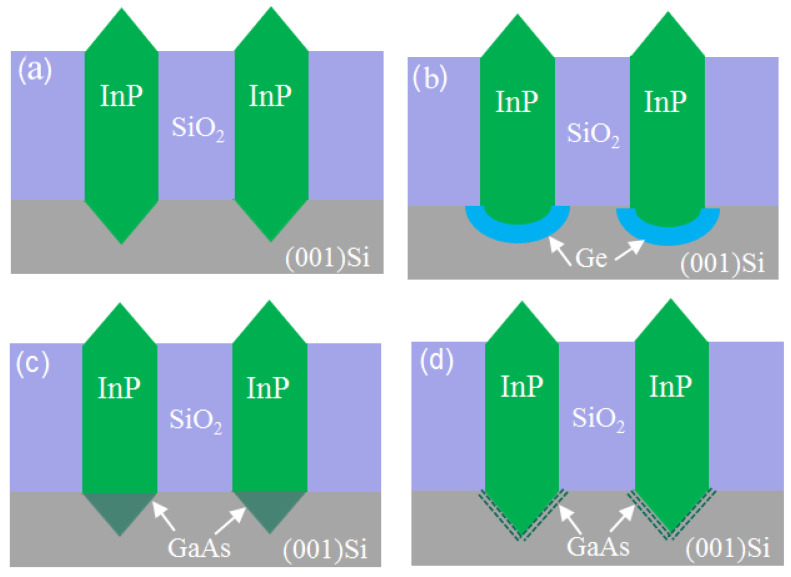
Four epitaxial schemes of InP on patterned Si using ART: (**a**) direct InP epitaxy on V-grooved Si; (**b**) InP on rounded Ge surface; (**c**) InP on a GaAs intermediate buffer filling up the V-grooves; (**d**) InP on a few–nanometer–thick GaAs stress relaxing layer.

In the III-V compounds’ semiconductor, InP global epitaxy on the Si substrate is rarely reported due to the difficulty of a huge 8% lattice mismatch. However, based on the advantages of ART technology, growing InP on silicon by the ART approach is common [132,133]. In the first place, the creation of {111}-oriented V-grooved pockets in trenches by this ART approach not only can prohibit the formation of APBs, but also promote strain relaxation via the formation of planar defects such as stacking faults. By designing the shape of the Si substrate, a highly twinned region is forming at the InP/Si interface, enabling a growth of active regions closer to the Si substrate. This twinned region is conducive to the strain relaxation when InP is deposited inside the V-grooved Si pockets. Moreover, InP can serve as a buffer layer for the growth of other III-V semiconductor compounds, such as InGaAs, InGaP. In the past few years, we summarize four growth schemes that were investigated by some researchers in Figure 26. Selective epitaxial growth of InP is deposited in different types of trench (V-grooved or rounded shape trench); different buffer layers (Ge, GaAs) are deposited for the growth of InP.

**Figure 27:**
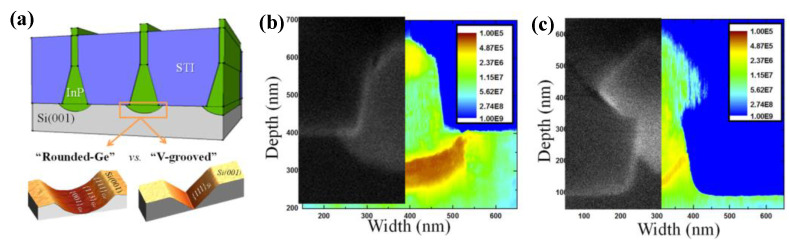
(**a**) Schematics of different starting surface (rounded-Ge and V-grooved Si) for the III-V growth on Si; Room temperature SSRM patterns in W = 80 nm trenches of InP epitaxially grown on **(b**) rounded-Ge and (**c**) V-groove Si starting surface. Reprinted with permission from ref. [134]. Copyright 2014 American Institute of Physics.

Although the creation of {111}-oriented V-grooved pockets in trenches by this ART approach can prohibit the formation of APBs, the weakness of this method is that it is impossible to capture the (111) steering defect along the parallel direction of the groove. This defect can result in stacking fault, twins on the sidewalls, in the upper InP layer, which makes it impossible to obtain large-size plane InP layer. Merckling et al. [134] studied the impact of starting geometry at the bottom STI on the crystalline alignment of the InP layer. They explored the starting geometry at the bottom as rounded etch with Ge buffer versus a crystalline <111> V-groove structure in the Si; the model is in Figure 27a. Rounded Ge structure exhibits different crystallographic facets such as {001}, {111}, and {113}, instead only {111} crystallographic planes on V-groove structure. Different crystallographic facets in rounded Ge surface may provide a nucleation surface with a unique polarity, which is inconducive to grow better nucleation uniformity. Instead, Si V-groove {111} enclosure will provide a surface with a unique polarity. The quality of InP layer was quantitatively characterized by HRXRD. The extracted FWHM value shows broad diffraction peak from InP grown on rounded Ge/Si at 1690 arcsec, while a much narrower diffraction peak from InP grown on “V” shape Si is 540 arc sec. Meanwhile, scanning spreading resistance microscopy (SSRM) was used to measure the local electrical resistance of InP for different process in Figure 27b,c. It is clear that low resistance (high conductivity) of the Ge buffer region is observed in the bottom of the trench, but a much thinner and a higher resistance of the V-groove InP/Si interface. Their SSRM results displayed that the InP grew on a rounded-Ge surface exhibits a low resistance (high conductivity) in the ~10^6^ ohms range in Figure 27b, while the resistance is clearly improved to ~5 × 10^7^ ohms range by using a V-groove starting surface in Figure 27c. In other words, higher quality of InP layer was achieved by the use of a V-grooved Si starting surface. In addition, more TDs were generating from the round Si/Ge interface, periodically decorated with misfit dislocations, propagating in the III-V layer.

**Figure 28:**
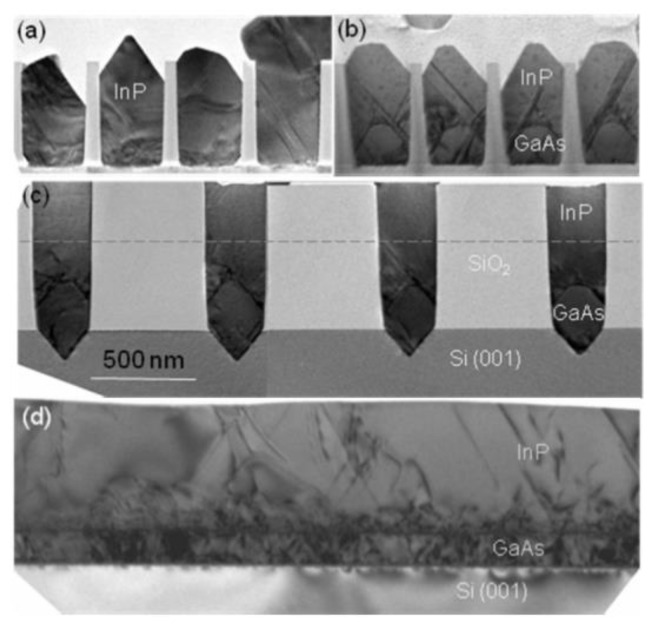
Cross-sectional TEM images of (**a**) InP/GaAs/Si with a 30 nm GaAs buffer layer; (**b**) InP/GaAs/Si with 200 nm intermediate GaAs layer; (**c**) InP/GaAs/Si structure with V-grooved Si surface and modified growth conditions and (**d**) same growth as (**c**) but grown on a Si (001) substrate. All images are to the same scale. Reprinted with permission from ref. [133]. Copyright 2009 IOP Publishing.

By mimicking the metamorphic InP/GaAs buffer on planar Si [135], GaAs and InP have the same crystal structure (sphalerite structure), which can reduce dislocation defects for the InP layer by using GaAs as the buffer layer. Figure 26c illustrates the model of a GaAs intermediate buffer layer. Li et al. [133] grew the high-quality uncoalesced thin films InP by MOCVD in SiO_2_ trenches on Si (001) via the ART method. Addition of a V-grooved Si surface to the ART process can more effectively trap misfit defects and APBs at a GaAs/Si intermediate interface as shown in Figure 28. They first directly grew InP in a blanket Si substrate with a different thickness of the GaAs layer. The 30-nm-GaAs buffer was not sufficient to allow misfit dislocations to be trapped in the ART structure. APBs and stacking faults were also observed from the Si surface to the upper InP layer as seen in Figure 28a. In Figure 28b, a thicker 200nm single GaAs buffer can make the stacking faults and twins originate from the InP/GaAs interface along {111}, which effectively inhibited the defects and APBs. Then, V-grooved surfaces at the trench bottom were formed for InP ART. A V-grooved feature would effectively increase the desired aspect ratio in the ART process and enable GaAs growth inside a pre-defined Si {111} enclosure, which can be expected to promote initial GaAs nucleation uniformity. From Figure 28c, it can be seen that high crystallographic quality of the InP is above the dotted line, where InP is essentially defect free. Figure 28d is the reference of InP global epitaxy on the exact (001) Si substrate, for which various mixed defects exist with high TDDs in the InP layer.

**Figure 29:**
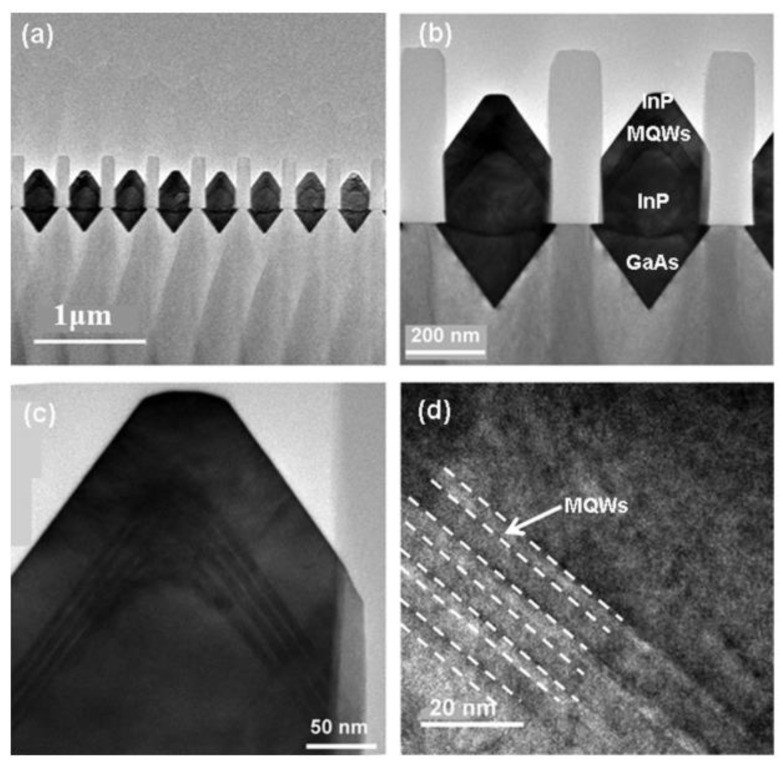
(**a**,**b**) Low magnification cross-sectional TEM image of an InGaAs/InP MQW grown on InP/GaAs buffer layers in nanoscale V-shaped trenches of Si (001) substrate; (**c**) A magnified cross-sectional TEM image of the top part of the sample; (**d**) A high-resolution TEM image taken from the region of side MQW. Reprinted with permission from ref. [136]. Copyright 2016 American Institute of Physics.

Bulk growth and quantum well (QW) are also induced as a dislocation filter to reduce TD in the ART technology further. Zhou [136] grew InP in nanoscale V-grooved trenches on the Si (001) substrate using InGaAs/InP multi-quantum-well by metal organic chemical vapor via the ART method. To obtain the best InGaAs quantum well potential barrier, a 60% In composition of InGaAs/InP MQW was deposited on InP/GaAs buffer layers in nanoscale V-grooved trenches. Figure 29 shows the cross-sectional TEM image of the sample. Highly uniform InGaAs/InP MQWs were visible over different trenches in Figure 29a. Few defects propagated through the GaAs/InP buffer layer to the MQW region in Figure 29b. From the magnification of InGaAs/InP MQW and the InP contact layer part, a clearly identified line and no crystalline defects are observed in Figure 29c. The four periods of 3 nm/6 nm InGaAs/InP QWs are observed with fine periodicity, flat and sharp interfaces in an atomic size, which indicated the highest quality of materials in Figure 29d.

**Figure 30:**
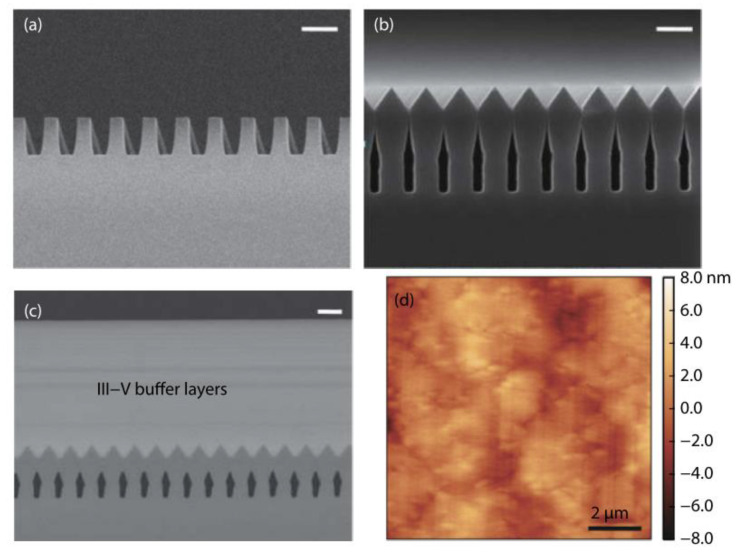
(**a**) Cross-sectional SEM images of U-shape patterned Si substrate; (**b**) Cross-sectional SEM images of homoepitaxy of 550 nm Si on (111)-faceted Si hollow; (**c**) III-V buffer layers on (111)-faceted Si hollow substrate; (**d**) AFM image of III-V buffer layers on Si substrate. Reprinted with permission from ref. [130]. Copyright 2019 IOP Publishing.

The use of {111}-oriented V-grooved pockets in SiO_2_ trenches by the ART approach is effective to restrict the generation of APBs and TDs, but the V-groove etched by the KOH solution is not only easy to cause serious damage to the crystal arrangement of the Si surface, but also easy to cause surface contamination by wet etching. In 2019, Wei et al. [130] grew the III-V materials on the V shape bottom of the {111} plane Si substrate by MBE. They first obtained the U-shaped pattern with ridges along [110] direction on the Si (001) substrate in Figure 30a; then, the homoepitaxy of the 550 nm Si layer was conducted by MBE at 600 °C. After that, the (111)-faceted Si hollow structures were achieved on the U-shape patterned Si (001) substrate in Figure 30b. The GaAs layers were deposited on the V shape Si substrate using a two-step method, as shown in Figure 30c. This outgrown (111)-faceted Si hollow structure is considered as a diatomic step to grow the ABP-Free GaAs layer on the GoVS template. More importantly, the thermal strain is released and attributed to this hollow structure. Figure 30d shows a perfect surface with the RMS at 1.3 nm and a low TDD of 7.0 × 10^6^ cm^–2^. This novel process can solve the problem of incompatibility between the miscut Si substrate and conventional Si substrate used in the CMOS process, which has a great commercial value.

From the above Figure 30, it is amazing that the presence of an extra free surface can absorb ~10% thermal strain between Si and GaAs layers, which can obviously reduce the TDD. As we know, the forming of the thermal crack is the thermal strain caused by the large CET of III-V and Si during the cool-down process. Controlling thermally induced strain during the growth is very important to the device fabrication. In order to prevent the crack formation, Saravanan et al. [137] grew GaAs films on a Si/porous Si/Si (SPS) substrate at a low temperature of 450 °C. Their result shows a biaxial tensile stress as low as 1.69 kbar for GaAs/SPS at the 77 K photoluminescence spectra, but with a higher TDD of 10^11^ cm^−2^. Inserting the buffer layer can decrease the TDD, while thicker buffer layers (~4 μm) can also induce thermal crack. Therefore, the strain compensated layer was introduced to reduce the thermal strain. Takano et al. [138] grew GaAs epilayers on Si substrates by inserting In_x_Ga_1−x_As buffer layers via low-pressure metalorganic vapor-phase epitaxy. The TDD of 4.8 × 10^6^ cm^2^ for the GaAs layer was achieved by insertion of an InGaAs strained layer. This incomplete strain relaxation in InGaAs layers can compensate the tensile strain due to the large TEC between GaAs and Si materials. However, the compositional buffer layer has some drawbacks, such as several microns in thickness, high cost, and large usage of material. In following, a thinner superlattice (SL) buffer was applied as the strain compensated layer to minimize the thermal crack, and the SL layer can not only facilitate strain relaxation by interaction with misfit dislocations, but also compensate the thermal strain energy from the mismatch of III-V to Si [107,116,136].

**Figure 31:**
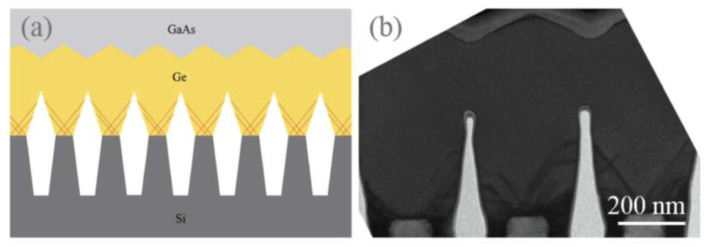

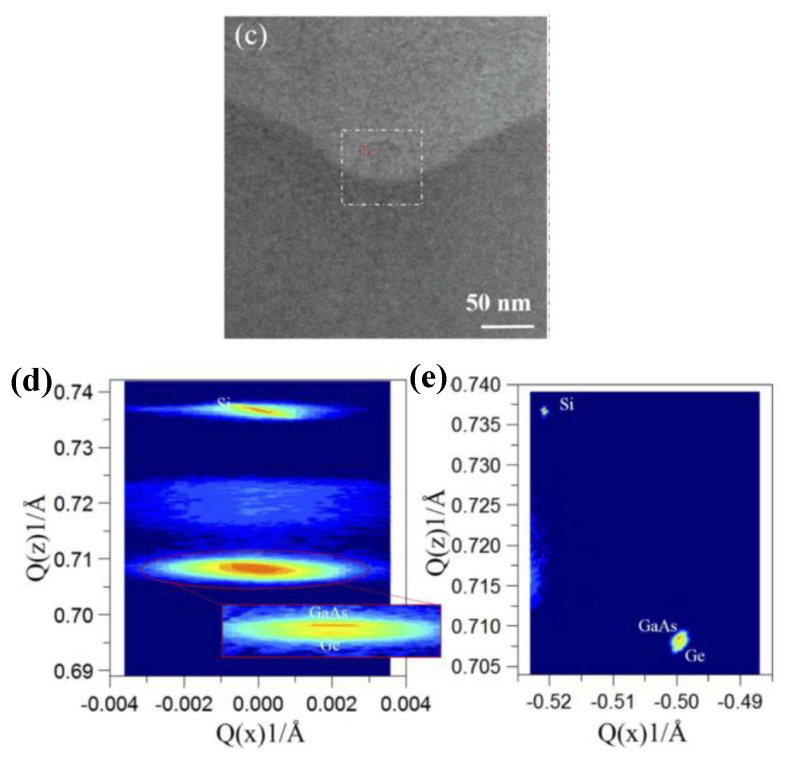
(**a**) The schematic illustration of the GaAs grown on the Ge/Si hollow substrate; (**b**) Cross-sectional TEM image of 600 nm thick Ge layer grown on the hollow patterned (001) Si; (**c**) Zoom-in HADDF image of the interface at the bottom region; high-resolution XRD reciprocal-space mapping around (**d**) (004) and (**e**) (224). Inset of (**c**) Higher resolution (004) map from triple-axis mode. Reprinted with permission from ref. [139].

Recently, the use of a patterned substrate to minimize the thermal crack was widely developed. As for pre-patterned growth, cracks can be avoided by growth on a small area due to strain relaxation near the pattern edge. As seen from Figure 31, the thermal strain of GaAs is released and attributed to this Si hollow structure; approaches using the Ge on the micropillar patterned Si (001) substrates were also proposed. Zhang et al. [139] grew APB-free GaAs film on employed the {113}-faceted Ge/Si (001) hollow substrate by MBE. First, the fabrication of the {113}-faceted Ge/Si (001) hollow substrate is as follows: the U-shape grating pattern was defined on an on-axis Si (001) substrate by using deep ultraviolet lithography and reactive ion etching; then, depositing the 60 nm Si buffer on the patterned substrate, a 600 nm Ge layer was grown to achieve the {113}-faceted Ge hollow structures, as shown in Figure 31a. Second, the typical two-step GaAs was grown on the {113}-faceted Ge/Si (001) hollow substrate in Figure 31b. Figure 31c shows the magnification of {113}-faceted Ge and the GaAs interface; few APBs were found at the bottom of the Ge {113} sawtooth structure, but APBs are not observed in the top GaAs layer. Similarly, the {113}-faceted Ge structure can annihilate the APBs at the interface with the {111}-faceted Si structure, which can be considered as a miscut substrate. However, the Ge {113} crystal plane has less miscut than the Si {111} crystal plane, which brings out a trapezoidal shape of APB in ten nanometers of the bottom of the Ge {113}. In following, the 7-µm-thick GaAs layer, which is far beyond the typical value of the cracking thickness, can be grown on the {113}-faceted Ge/Si hollow substrate. They characterized the thermal strain issue by a high-resolution XRD reciprocal space mapping (RSM) performed around (004) and (224) reflections, as shown in Figure 31d,e. From the peak position of RSM, the in-plane strain ε_‖_ of the GaAs and Ge layer were calculated from the extracted calculation of the in-plane lattice constant and out-of-plane lattice constant of the GaAs and Ge. Compared with the lattice constant of bulk GaAs and Ge, the residual thermal strain of Ge is about 89.8% lower than that of the Ge layer on normal Si substrates [140]. The residual thermal strain of GaAs is 29.4% lower than that of the GaAs layer grown on the Ge/Si template [141]. Their results indicated that this hollow structure plays an essential role in thermal strain reduction. With a 400 nm GaAs deposition, a smooth GaAs surface with a RMS of 0.67 nm was acquired, and a low TDD of 5.7 × 10^6^/cm^2^ was obtained by following InGaAs/GaAs quantum-well DFLs inserting.

#### 4.2.2. Epitaxial Lateral Overgrowth (ELO)

**Figure 32:**
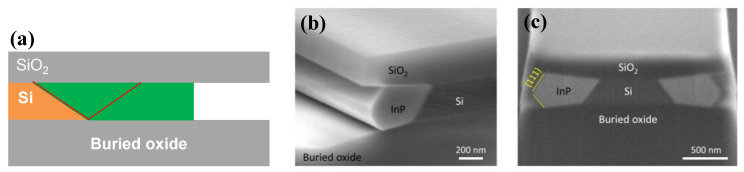
(**a**) The scheme of lateral ART. The red dotted lines denote the confinement of the majority of crystalline defects at the InP/Si interface and the trapping of residual TDs by the top and bottom SiO_2_ layers. (**b**) Tilted view SEM image of one InP sandwiched between the top oxide spacer and the buried oxide layer. (**c**) Cross-sectional SEM images of two symmetrical InPs grown using the lateral ART approach. Reproduced with permission from ref. [143]. Copyright 2019 American Institute of Physics.

**Figure 33:**
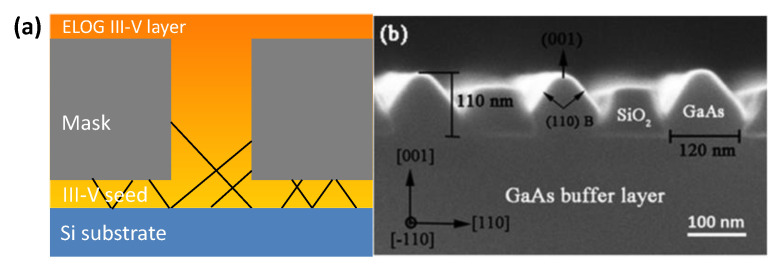

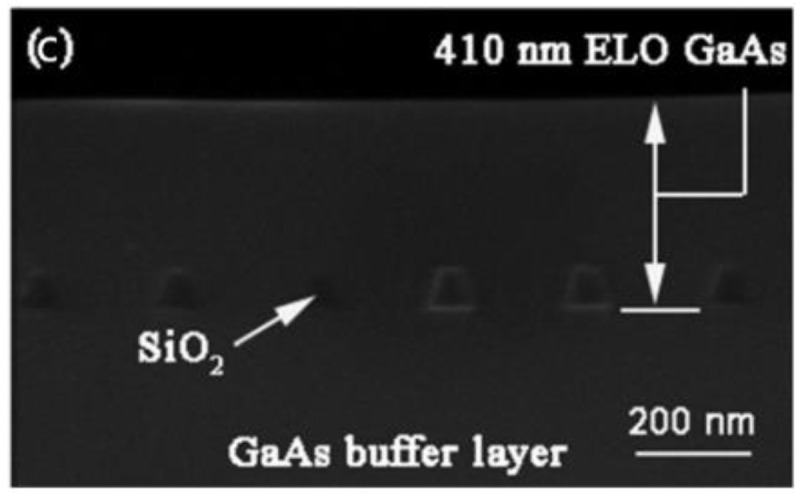
(**a**). Schematics of lateral epitaxial overgrowth (ELO); (**b**) cross-sectional SEM images of the films after the first selective growth stage; (**c**) cross-sectional SEM images of the ELO GaAs layer. Reprinted with permission from ref. [147]. Copyright 2015 American Institute of Physics.

Although the ART technique discussed above can effectively solve the APB problem, and block the propagation of crystalline defects using the groove mask, this technique also limits the maximum achievable dimension of III-V epitaxial layers, which are more amenable in some device applications [142]. In order to provide a large film plane for the preparation of III−V devices, the ART technique needs to be continuously optimized so that the SEG III−V materials are epitaxial and grown out of closely spaced trenches, and extend laterally above the oxidation strips until the materials from adjacent grooves merge together to form a continuous high quality epitaxial layer. Recently, an alternative ART technique called Lateral aspect ratio trapping (LART) was developed. As schematically illustrated in Figure 32a, micrometer-scale III-V crystals can be directly grown above the buried oxide layer by this LART method [143]. Compared to the conventional “aspect ratio trapping” approach, the LART approach is changing the direction of the groove, enabling the growth front to the lateral direction. The lateral oxide trenches can be created by dry etch terminated on the buried oxide; then, anisotropic wet etching was processed to form “V” shaped Si. This {111}-oriented Si bevel structure not only prohibits the formation of APBs in the initiating lateral growth of InP layers, but also the propagation of TDD was effectively blocked by the wide lateral SiO_2_ trenches, which reveal a high “aspect ratio” in the lateral direction. Figure 32b,c shows a tilted-view SEM image of the SEG InP layer grown on (001) SOI substrates using the LART method. Figure 33b shows an InP-epi “wing” grown using the lateral ART approach, and Figure 33c displays the two symmetrical InP-epi wings. From the SEM images, we can see the Si pedestal sandwiched between the top oxide spacer, and the buried oxide layer features two {111}-oriented surfaces. Starting from the nucleation sites provided by the {111} Si facets, the InP crystal evolves laterally along the [110] direction into wing-structures with two {111} facets. The angle between the two {111} facets is around 110° which indicates a zincblende crystal structure Then, the InP stripes continue laterally to grow inside the long nano-scale SOI trenches resulting in dislocation-free InP crystals right atop the buried oxide layer. In addition, the dimension of III-V nano-ridges can be controlled by changing the thickness of the Si layer on SOI, which is limited by the size of photolithography in the conventional ART approach. This in-plane and close placement of the III-V layer with the Si device layer also facilitates the integration of III-V light emitters with Si-based photonic components.

The above ART methods or lateral ART approach often produce sub-micrometer dimensions of APB-III-V layers, but to expand the dimension of the epitaxial III-V further to tens and even hundreds of micrometers, ART technology needs to be improved. Epitaxial lateral overgrowth (ELO) is an innovative technique to provide large regions of device materials. In the ELO process, a III-V buffer layer is first grown on the Si substrate, then polished by CMP. Microsized SiO_2_ stripe patterns are selectively etched to expose the III-V buffer layer for regrowth. Then, the III-V epitaxial layer is vertically regrown through the opened region of the mask and thereafter can be laterally grown over the mask. Most of TDs in the buffer layer are blocked by the bottom of the mask, but a small number of TDs around the opened region will propagate upwards, as shown in Figure 33a. Therefore, a high crystalline quality and thicker layer can be made by this ELO. For the GaAs on Si, there are many pioneering works reported [144,145]. Tsaur et al. [146] first obtained the single-crystal GaAs layers by means of ELO seeded within stripe openings in a SiO_2_ mask over GaAs layers grown on Ge-coated Si substrates. TEM and scanning cathodoluminescence studies indicated that the laterally overgrown GaAs layers have a dislocation density. However, the early reports on ELO for GaAs-on-Si suffered from the limited defect-free region and the mechanical weakness of the laterally grown parts, both of which became severe as the ELO layer was further grown. In 2015, Yunrui et al. [147] demonstrated that the GaAs coalescence layer grew on the patterned 1.8 μm GaAs buffer layer by epitaxial lateral overgrowth using metal-organic chemical vapor deposition. A 410-nm-thick coalesced ELO GaAs layer was obtained with the low root-mean-square surface roughness of 6.29 nm. Figure 33b,c show the SEM images of the growth and evolution of the ELO GaAs layer on the opening trenches. As shown in Figure 33b, the growth front of GaAs is faceted, with (111) B on both sides and (001) on the top. With the deposition time, the overall 410 nm ELO GaAs layer was shown in Figure 33c.

**Figure 34:**
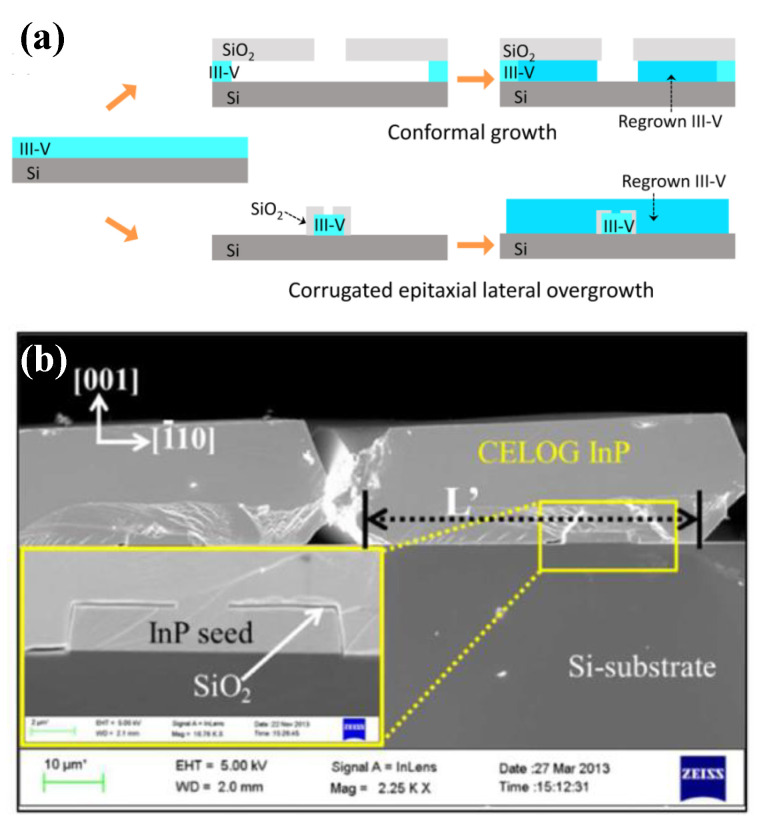
(**a**) Schematic of two improved ELO process; **(b**) cross-sectional SEM in [110] direction view of CELOG InP on Si. Reprinted with permission from ref. [154]. Copyright 2015 IOP Publishing.

Compared with GaAs-on-Si, ELO techniques are more widely employed [148,149,150] on InP-on-Si growth, because it is very difficult to obtain low TDDs and large-scale via the direct growth of InP on Si. For the optimization of InP ELO on the (001) Si substrate, Metaferia et al. [151] investigated the ELO of InP from mesh and line openings on the masked InP seed layer on the Si (001) wafer. Their results showed that the coalesced region produced the TDD in a range from 6 × 10^6^ cm^−2^ to 4 × 10^7^ cm^−2^ depending on the thickness of the ELO layer. In their work, the ELO InP layer was grown on a 1.5-µm-thick InP/Si substrate with a 40-nm-thick SiO_2_ mask. The mesh and line masks (opening and masking width of 200 nm and 3 µm, respectively), tilted 15°and 30°off the [110] direction, are compared. The quicker coalescence in the mesh opening resulted in better surface roughness at the early growth stage (~10-µm-thick InP layer), but, after an extended growth (~100 µm-thick InP layer), both mesh and line opening cases exhibited a similar surface roughness (RMS roughness of 16~25 nm). It was shown that regardless of both angles (15° and 30°) and masks (mesh and line opening), the TDDs were measured to be a similar value. The TDD of the 10 µm-thick and 100 µm-thick InP layer was measured to be 2~4 × 10^7^ cm ^−2^ and 6~7 × 10^6^ cm^−2^, respectively. Han et al. [152] displayed two improved ELO strategies to achieve large-dimension III-V materials with a close proximity to the Si substrates in Figure 34. The first scheme is called conformal growth; III-V films lateral selective epitaxial growth starts from III-V seeds on silicon. In the conformal growth scheme [153], after the III-V films were deposited on the Si substrate, a thin layer of the growth mask is then patterned to form the III-V seeds. Then, the regrown III-V stripes were laterally selectively grown along the initial III-V seeds to extend the large dimension size. Another scheme is named corrugated epitaxial lateral overgrowth (CELO) [154]; short III-V thin film segments were processed as a III-V seeds by bulk III-V film deposition and patterned; then, a thin patterned oxide mask was deposited on III-V seeds for the following homogenous selective regrowth of the InP process. In both strategies, crystalline defects are confined within the seed mesa, while the laterally overgrown III-V is free of any TDs. Figure 34b shows the cross-sectional SEM in [110] view of CELOG InP on Si; defects are confined in the lateral trenches, and no TDS can be found in the CELOG InP layer. These two improved processes can be used for large-dimension III-V layer manufacturing, which could be furthered processed to the subsequent growth of lasers’ structures.

**Figure 35:**
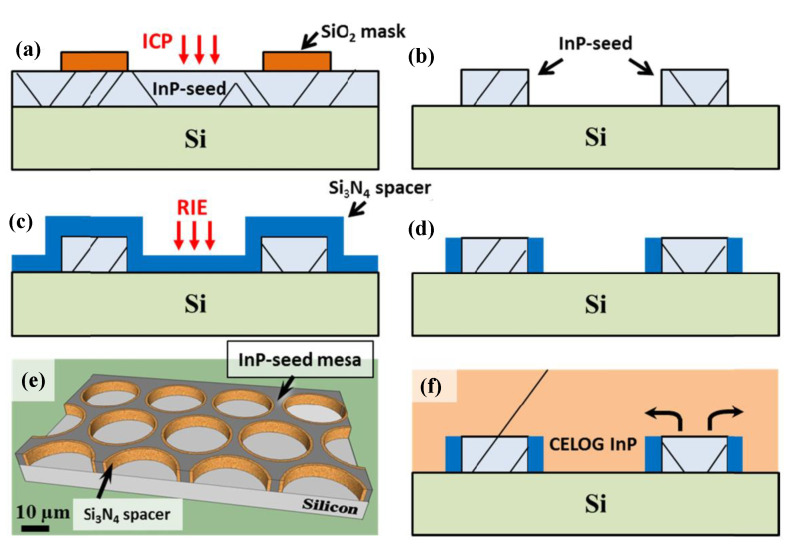
(**a**–**d**) Process of InP-seed mesa fabrication for CELO; (**e**) Schematic of the InP-seed mesa pattern on Si before CELO. The thickness of InP-seed layer is 2 μm, and the distance between the two adjacent circular rings of diameter 30 μm is 5 μm; (**f**) Schematic of the CELO InP/Si cross-section. Reprinted with permission from ref. [155].

Following the above results, in 2020, Omanakuttan et al. [155] fabricated and studied the high crystalline quality of InP by the self-aligned corrugated epitaxial lateral overgrowth (CELOG) method. Figure 35a–d display the InP-seed mesa fabrication process flow. A schematic of the InP-seed mesa was patterned on Si processed for CELOG and is shown in Figure 35e. Through a series of processes: mask deposition, photolithography, and etching, then the CELOG of InP/Si can be obtained by a schematic of Figure 35f. The defective InP seed acts as the CELOG InP growth origination; the threading dislocations propagating from the InP seed can be eliminated by creating dislocation loops for increasing the InP layer thickness. Their results displayed that RMS surface roughness of 2.95 nm obtained for the uniform InP CELOG layer. A higher intensity band edge emission in the cathodoluminescence spectra near-BE band at 892 nm (1.39 eV) and enhanced carrier lifetime (710 ps) of InP are observed above the CELO InP/Si interface compared to the defective seed InP layer on Si, which are attributed to the reduced TDD realized (~3 × 10^8^ cm^−2^) by the CELO method. For the application of the photonic integrated circuit (PIC), a large dimension and uniform InP layer with ultralow dislocation on Si are desired. The CELOG can be extended to the formation of other large dimensions of III-V/Si heterostructures.

In the laterally grown parts, propagating defects such as threading dislocations and stacking faults will be blocked by the mask and consequently cannot propagate into the layer above the mask. ELOG was used to grow GaN [156], as well as InGaAs [157] on Si.

**Figure 36:**
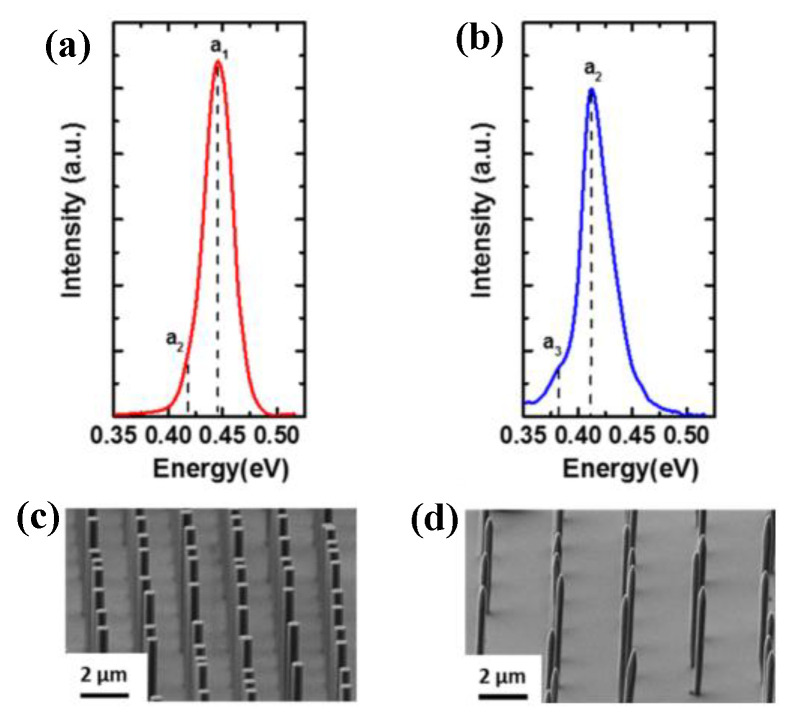
PL spectra at 10 K of InAs NWs grown on (**a**) GaAs (111) B and (**b**) Si (111); (**c**,**d**) Corresponding SEM images. The pitches are, respectively, 2 and 2.5 μm. Reprinted with permission from ref. [162]. Copyright 2021 American Chemical Society.

The InAs semiconductor material is a very good candidate for ultra-high-speed electronic and optoelectronic devices, due to its narrow band gap, small electron effective mass, and very high electron mobility (~33,000 cm^2^/V s at 300 K). However, the large epitaxial strain generated by the high lattice mismatch (11.4%) and the difference in thermal expansion between InAs and Si, leads to a roughness surface and high TDD, which degrade the electrical and optical properties. At present, growth of thin-film InAs is usually difficult, but direct growth of vertical nanowires can usually accommodate a larger lattice mismatch as compared to thin-film growth [158]. Regarding InAs nanowires, various strategies including vertical VLS growth [159], vertical SEG growth [160,161] have been proposed. Among these techniques, vertical VLS growth has an obvious disadvantage: using Au as the catalyst leads to the formation of deep-level traps with silicon and degrades the device performance, which makes it forbidden in the CMOS fabrication process, so vertical SAE growth was widely developed. In the process of InAs NWs’ growth, the morphology of NWs is essential to homogeneous optical and electrical properties. Hertenberge et al. [160] obtained very high yields of ~90 percent of vertically (111)-oriented InAs nanowires selectively grown on the patterned Si(111) substrate. Then, Bjork et al. [161] studied (111)Si (axial) and (1-10)Si (radial) growth of InAs NWs by varying growth duration, temperature, group-III molar flows, V/III ratio, mask material, mask opening size, and inter-wire distance. To achieve uniform nucleation and a high vertical yield of wires, an As-terminated surface and an optimized TMIn flow and V/III ratio are required. Below 520 °C and 540 °C, respectively, the <111> and the <1-10> growth is surface kinetically limited. Their results also indicated that by placing wires in large arrays, it is possible to stop the <1-10> growth rate completely in favor of the <111> growth rate. Recently, Grégoire et al. [162] report that the vertical and high aspect ratio InAs NWs with a hexagonal shape were grown on both GaAs (111)B and Si(111) patterned substrates by selective epitaxial growth (SEG). The morphology and the quality of InAs NWs’ arrays grown on GaAs(111)B and Si(111) were characterized by SEM and photoluminescence measurements in Figure 36a–d, respectively. For NWs grown on GaAs(111)B in Figure 36a, a strong peak at 0.445 eV with a full width at half-maximum (FWMH) of 32meV was observed, while a low peak at 0.413 eV with a FWHM of 34 meV grown on Si was observed, as shown in Figure 36b. A lower PL intensity recorded from InAs NWs grown on Si compared to GaAs is probably due to the lower NW density and diameter. By decreasing the NW diameter, the principal PL peak shifted to a higher energy, confirming the presence of both WZ and ZB phases in InAs NWs grown by SAG-HVPE. These NW arrays exhibited strong PL intensity and optical absorption, which is encouraging for future optical devices.

**Figure 37:**
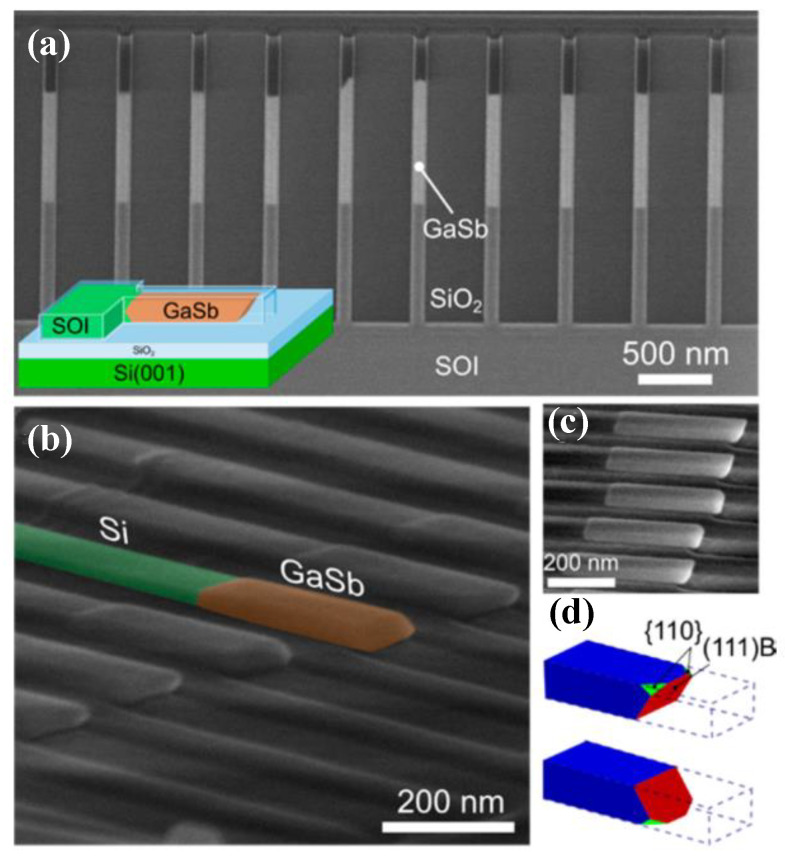
(**a**) SEM image of an array of horizontal GaSb nanostructures integrated coplanar with a SOI layer using TASE. The inset shows an illustration of the layer structure. (**b**,**c**) Tilted SEM images of GaSb nanostructures. (**d**) Schematic illustration highlighting the typical faceting observed at the growth front of the GaSb crystals. Reprinted from ref. [167]. Copyright 2017 American Chemical Society.

In the III−V semiconductor, III-antimonide, such as GaSb, has attracted a tremendous amount of research interest. GaSb (0.72 eV) is a direct bandgap material, which can be used as a photodetector of the mid-IR spectrum. Moreover, GaSb exhibited attractive characteristics for p-MOSFETs due to their high bulk hole mobility (over 1000 cm^2^/Vs). Similarly, it has great challenges to grow GaSb on Si substrate, due to the large lattice mismatch and thermal mismatch. Recently, there were reports of a GaSb-on-insulator (-OI) by direct wafer bonding [163] and epitaxial layer transfer [164], but these methods remain costly due to the limited sizes of available III−V substrates. However, a one-dimensional (1D) GaSb nanowire (NW) manufactured by selective epitaxial growth (SEG) provides extra benefits for many advanced utilizations, such as the better stress relaxation, capability of the advanced gate stacking integration, more efficient light adsorption and trapping. For the GaSb NWs’ application in electronic devices, a well-controlled nanowire-like morphology with a small diameter (several tens of nanometers) is essential. Yang et al. [165] grew very thin and uniform GaSb NWs with diameters down to 20 nm by the use of a sulfur surfactant. This GaSb NWs were configured into transistors and exhibited impressive electrical properties with the peak hole mobility of ~200 cm^2^/Vs, better than any mobility value reported for a GaSb nanowire device. To control the GaSb crystal shape and dimension deeply, an alternative to the SEG technique known as template-assisted selective epitaxy (TASE) was developed [166,167]. Borg et al. [167] investigated a monolithic integration of high-mobility horizontal GaSb NWs on the SOI substrate by TASE. They found that a high degree of morphological control allows for GaSb nanostructures with critical dimensions down to 20 nm. Figure 37a displays a SEM image of an exemplary array of GaSb nanostructures outside the templates. The exposed front GaSb surface, at which the crystal grows, typically forms a large {111} facet often with two smaller and opposing {110} facets (see Figure 37c,d). Because of the polar nature of the GaSb zinc-blende crystal structure, nonequivalent 180° rotations of the crystal lattice can occur upon nucleation on the nonpolar Si. This results in two distinct orientations of the growth facets as illustrated in Figure 37d. Meanwhile, the GaSb growth is governed by excess Sb present on the GaSb surface, leading to a strong growth rate dependence on the V/III ratio and temperature. Hall/van der Pauw measurements are conducted on 20-nm-thick GaSb nanostructures, revealing high hole mobility of 760 cm^2^/(V s).

In summary, the APB problem can be solved by a miscut Ge/Si substrate, {113}-faceted Ge/Si (001) hollow substrate in the global epitaxial method, and {111}-oriented V-grooved in the ART method. However, high quality III−V heteroepitaxy with low TDD on Si is crucial to the monolithic integration of III−V devices on Si-based PICs. Table 2 is the summary of the reported TDD and RMS values of GaAs, InP in the growth of the substrate, epitaxy method, buffer materials, process in these years. In terms of material growth quality, the TDD of the GaAs layer as low as 10^7^ cm^−2^ can be achieved by inserting the superlattice via global epitaxy. Then, the 8-inch GaAS-O-I substrate can be realized in our group soon. However, few research projects report on the preparation of other III−V epitaxy methods on the Si substrate. For selective epitaxial growth (SEG), many III−V semiconductors such as InP, InCaP, InAs, GaSb, etc. can be grown by the ART method. A TDD of 10^6^ cm^−2^ of the InP layer was obtained in the V-grooves patterned Si substrate. In terms of application, the high-quality and stress-controlled large-size III-V epitaxial layer grown by global epitaxy is more conducive to the preparation of Si-based OEIC. On the contrary, the III-V nanowire arrays fabricated by the SEG method can produce the monolithic integration of III-V nanodevices on silicon substrates. Especially for a smaller size of CMOS electronic devices, several tens of nanometers of III-V NWs are considered optimal for high-performance tunnel-FETs.

## 5. Conclusions and Outlooks

High-quality III-V heteroepitaxy on the Si substrate is crucial to the Si-based optoelectronic integration circuits (OEICs). In this work, we reviewed the three major challenges of the Si based IIII-V heteroepitaxy: (1) anti-phase boundaries (APB); threading dislocation (TD); (3) stacking faults (SF). Meanwhile, the mechanism and theoretical model of three kinds of defects are also deeply analyzed, which is convenient for readers to understand its development process in detail. In order to solve each issue, a wide variety of strategies is discussed. The bulk and high quality of III-V structure layers are the basis of larger scale optoelectronic devices’ fabricating. For global epitaxy, the offcut Si substrates can easily suppress the formation of APBs, but it is incompatible with the integration of CMOS manufacturing. To reduce the TDDs, a lot of methods, including the buffer layer, annealing, multi-step growth, defect filter layers, and so on, were developed. In this, the thick layer and high annealing process can lead the new defects as stacking faults and thermal cracks, resulting in the low TDD of 10^6^ cm^−2^ for GaAs, and a higher TDD (~10^7^ cm^−2^) of InP-on-Si due to a larger lattice mismatch (~8%) between InP and Si. For selective epitaxial growth (SEG), {111}-oriented V-grooved pockets in SiO_2_ trenches by the ART approach are effective to restrict the generation of APBs and TDs, but the solution-etched V-groove is not always uniform and easily damages the crystal arrangement of the Si surface, which obviously affects the defects’ distribution. In addition, the ART method also limits the maximum achievable dimension of the epitaxial III-V materials. Epitaxial lateral overgrowth (ELO) is an innovative technique to provide large regions of device materials, especially for plane-InP growth on Si. Moreover, other III-V semiconductors, such as InAs, GaSb, which have high mobility but larger mismatches with the Si substrate, can be fabricated in small-size Nanowires (NWs) by the ART method, representing potential applications as the channel material integrated on Si substrates to MOSFETs devices.

Even though major obstacles for III-V-on-Si heteroepitaxy, such as APBs, TD, stacking faults, are resolved now, the quality of III-V layers on on-axis Si is still unsatisfactory for the deployment on the PICs or ICs. Currently, we can obtain the low TDD of 10^6^ cm^−2^, but it still challenging to reduce the TDD to native substrates (~10^4^ cm^−2^). Therefore, further effects need to be devoted to improving the quality of the III-V layer on Si to realize the integrated III-V on the Si-based platform fully. The Epitaxial Lateral Overgrowth (CELO) is an innovative process for large-scale high-quality Si-based III-V epitaxial layers; systematic research and process optimization are still needed to improve the materials’ quality and device performance further. In addition, the bounding of III-V-O-I can thin the LT low-quality layer, which can fabricate the high-performance optoelectronic devices. Therefore, based on the current research, efforts should continue to be devoted to resolving the TDDs and the compatibility of integration techniques with large-volume CMOS manufacturing.

## Figures and Tables

**Table 1:** Different characteristic data of GaAs/Si samples grown with different procedures. Reprinted with permission from ref. [105] Copyright 2013 American Vacuum Society.

Samples	Growth Procedure	DCXRD ω−Scan FWHM (arcsec)	RMS Roughness in 10 × 10 μm^2^ (nm)	TDD (cm^−2^)
A1	Two−step growth	327.5	2.9	4.4 × 10^7^
A2	Two−step growth + one TCA step	210.2	2.5	1.9 × 10^7^
B1	Three−step growth	298.2	2.4	3.7 × 10^7^
B2	Three−step growth + one TCA step	184.3	2.0	1.4 × 10^7^
B3	Three−step growth + two TCA steps	164.2	1.8	1.1 × 10^7^

**Table 2:** Summary of reported TDD value of III-V materials in terms of substrate, epitaxy method, buffer, procedure.

Year	Substrate	Epitaxy Method	Buffer	Procedure	III-V Materials	TDD (cm^−2^)	RMS (nm)	Refs.
2011	0° Si	Global	Ge	two-step growth	GaAs	2 × 10^7^	1.1	[70]
2011	4° Si	Global	Ge	two-step growth	GaAs	1.8 × 10^7^	―	[64]
2013	0° Si	Global	―	three-step growth+ TCA	GaAs	1.1 × 10^7^	0.73	[113]
2014	Ge	Global	―	two-step growth	GaA	2.7 × 10^7^	0.7	[93]
2015	6° Ge	Global	―	two-step growth	GaAs	―	0.6	[97]
2016	0° Si	Global	Ge	two-step growth	GaAs	3 × 10^7^	0.5	[98]
2018	0° Si	Global	Ge	In_0.18_Ga_0.82_As/GaAs SLSs	GaAs	2.3 × 10^6^	―	[121]
2019	0° Si	ART	Si	(111)-faceted Si hollow	GaAs	7.0 × 10 ^6^	1.3	[138]
2020	0° Si	ART	Ge	{113}-faceted Ge/Si hollow substrate.	GaAs	5.7 × 10^6^	0.67	[145]
2021	0° Si	Global	CMP-Ge	three-step growth	GaAs	7.4 × 10^7^	1.27	[53]
2018	0° Si	Global	Ge	InAs/InP QD DFLs	InP	3 × 10^8^	2.88	[127]
2011	0° Si	ELO	―	two-step growth	InP	4 × 10^8^	―	[158]
2019	0° Si	CELOG	―	two-step growth	InP	3 × 10^8^	2.95	[162]
2020	0° Si	Global	―	In_0.73_Ga_0.27_As/InP SLSs	InP	4.5 × 10^7^	2.38	[115]
